# Combined Targeted and Untargeted Profiling of HeLa Cells Deficient in Purine De Novo Synthesis

**DOI:** 10.3390/metabo12030241

**Published:** 2022-03-13

**Authors:** Lucie Mádrová, Olga Součková, Radana Brumarová, Dana Dobešová, Jan Václavík, Štěpán Kouřil, Julie de Sousa, Jaroslava Friedecká, David Friedecký, Veronika Barešová, Marie Zikánová, Tomáš Adam

**Affiliations:** 1 Institute of Molecular and Translational Medicine, Faculty of Medicine and Dentistry, Palacký University and University Hospital in Olomouc, Hněvotínská 1333/5, 779 00 Olomouc, Czech Republic; luca.madrova@gmail.com (L.M.); radana.karlikova@gmail.com (R.B.); dobesova.dana147@gmail.com (D.D.); janvaclavik87@gmail.com (J.V.); julie.jdesousa@gmail.com (J.d.S.); jaroslava.jacova@gmail.com (J.F.); david.friedecky@upol.cz (D.F.); 2 Department of Paediatrics and Inherited Metabolic Disorders, First Faculty of Medicine, Charles University and General University Hospital in Prague, Ke Karlovu 455/2, 128 08 Prague, Czech Republic; olga.souckova@lf1.cuni.cz (O.S.); veronika.baresova@lf1.cuni.cz (V.B.); marie.zikanova@lf1.cuni.cz (M.Z.); 3 Department of Clinical Biochemistry, University Hospital in Olomouc, I. P. Pavlova 6, 775 20 Olomouc, Czech Republic; kourilstepan@gmail.com; 4 Department of Mathematical Analysis and Applications of Mathematics, Palacký University Olomouc, 17. listopadu 12, 771 46 Olomouc, Czech Republic; 5 Institute of Molecular and Translational Medicine, Czech Advanced Technology and Research Institute (CATRIN), Palacký University Olomouc, Hněvotínská 1333/5, 779 00 Olomouc, Czech Republic

**Keywords:** rare metabolic disorders, purine de novo synthesis, HeLa cells, metabolomics, mass spectrometry

## Abstract

Three genetically determined enzyme defects of purine de novo synthesis (PDNS) have been identified so far in humans: adenylosuccinate lyase (ADSL) deficiency, 5-amino-4-imidazole carboxamide-ribosiduria (AICA-ribosiduria), and deficiency in bifunctional enzyme phosphoribosylaminoimidazole carboxylase and phosphoribosylaminoimidazolesuccinocarboxamide synthase (PAICS). Clinical signs of these defects are mainly neurological, such as seizures, psychomotor retardation, epilepsy, autistic features, etc. This work aims to describe the metabolic changes of CRISPR-Cas9 genome-edited HeLa cells deficient in the individual steps of PDNS to better understand known and potential defects of the pathway in humans. High-performance liquid chromatography coupled with mass spectrometry was used for both targeted and untargeted metabolomic analyses. The statistically significant features from the untargeted study were identified by fragmentation analysis. Data from the targeted analysis were processed in Cytoscape software to visualize the most affected metabolic pathways. Statistical significance of PDNS intermediates preceding deficient enzymes was the highest (*p*-values 10 × 10^−7^–10 × 10^−15^) in comparison with the metabolites from other pathways (*p*-values of up to 10 × 10^−7^). Disturbed PDNS resulted in an altered pool of adenine and guanine nucleotides. However, the adenylate energy charge was not different from controls. Different profiles of acylcarnitines observed among deficient cell lines might be associated with a specific enzyme deficiency rather than global changes related to the PDNS pathway. Changes detected in one-carbon metabolism might reduce the methylation activity of the deficient cells, thus affecting the modification state of DNA, RNA, and proteins.

## 1. Introduction

Purines are heterocyclic imidazolopyrimidine molecules that are essential for a multitude of basic functions in cell physiology. They are the vital building blocks of nucleic acids, energy intermediates, signaling molecules, allosteric modulators, and coenzymes. Hence, they play a central role in metabolic regulation and energy homeostasis, as well as taking part in many reactions of signal transduction and translation, the cell cycle, immune response, and others. A highly regulated balance between purine de novo synthesis (PDNS), recycling, and degradation is responsible for maintaining a pool of purine nucleotides according to cellular needs [1]. The cell uses preferential recycling of purine bases for its normal growth. Under conditions such as the G1 and S phases of the cell cycle, the high need for purines makes the cells use PDNS predominantly [2,3].

Biosynthesis of purines de novo is an energetically demanding metabolic pathway that uses six enzymes (some multifunctional) to convert 5-phosphoribosyl-1-pyrophosphate (PRPP) to inosine-5-monophosphate (IMP) in ten chemical reactions. Besides five molecules of adenosine-5-triphosphate (ATP), one molecule each of glycine, aspartate, and carbon dioxide, and two molecules each of formate and glutamine are needed for the generation of a molecule of IMP. In 2008, using fluorescent microscopy, the six enzymes involved in PDNS were found to form a macromolecular complex under purine-depleted conditions. This newly discovered metabolon was termed a purinosome and has been intensively studied since [2]. The core of purinosome is formed by phosphoribosyl pyrophosphate amidotransferase (PPAT, EC 2.4.2.14), GART-trifunctional enzyme phosphoribosylglycinamide synthetase (EC 6.3.4.13)/phosphoribosylglycinamide formyltransferase (EC 2.1.2.2)/phosphoribosylaminoimidazole synthetase (EC 6.3.3.1) and phosphoribosylformylglycinamidine synthase (PFAS, EC 6.3.5.3), whereas bifunctional PAICS (phosphoribosylaminoimidazole carboxylase, EC 4.1.1.21; phosphoribosylaminoimidazolesuccinocarboxamide synthase, EC 6.3.2.6), ADSL (EC 4.3.2.2), and ATIC-bifunctional enzyme composed of phosphoribosylaminoimidazolecarboxamide formyltransferase (EC 2.1.2.3) and IMP cyclohydrolase (EC 3.5.4.10) which act on the periphery [4]. The formation of purinosome was found to be microtubule-assisted in HeLa cells [5]. Super-resolution fluorescent microscopy revealed that purinosomes also colocalize with mitochondria [6], and several hypotheses have been postulated since to explain this behavior [7]. Recently, channeling of metabolites in PDNS within the purinosome in situ was visualized by three-dimensional sub-micrometer chemical imaging of single cells (HeLa) by gas cluster ion beam mass spectrometry (GCIB-SIMS) [8]. For efficient production of purines, the enzymes of PDNS are regulated by several mechanisms such as feedback inhibition, changing the oligomeric state of an enzyme, regulation by heat shock proteins 70 and 90, casein kinase 2, and the PI3K/AKT/mTOR signaling pathway [9,10,11,12,13,14].

Three genetic defects of the pathway resulting in decreased enzymatic activity have been identified in humans so far, all of them extremely rare. AICA-ribosiduria (ATIC deficiency, OMIM 608688) is caused by mutations in the *ATIC* gene encoding for AICARTF (AICAR transformylase) and IMPCH, which catalyzes the last two reactions of PDNS. Only four patients have been diagnosed to date, presenting with an accumulation of 5-amino-4-imidazolecarboxamide riboside (AICAr), succinyl aminoimidazole carboxamide riboside (SAICAr), and succinyladenosine (S-Ado) in body fluids [15,16]. Mutations in *ADSL* gene cause ADSL deficiency (OMIM 103050) characterized by high levels of SAICAr and S-Ado in body fluids. Unlike for AICA-ribosiduria, more patients (almost 100) have been identified so far [17], showing considerable variations in clinical signs according to different ratios of accumulating metabolites [18]. These include seizures, delayed growth, psychomotor retardation, autistic features, hypotonia, and others [19]. In 2019, PAICS deficiency was identified by genetic analysis of two siblings who died early after birth due to multiple malformations. The accumulation of AIr in patient body fluids was not measured due to their unavailability [20]. Thus far, functional mutations in GART or PFAS have not been reported. Diverse clinical presentation, underdiagnosing due to relatively demanding laboratory analyses, and limited awareness of genetic defects in PDNS might be the reason for an underestimated prevalence rate of these diseases [21,22].

In general, profound defects in PDNS are presumably fatal for embryonic development. The exact mechanism by which a particular enzyme deficiency of PDNS leads to a clinical outcome remains unresolved. Except for a potential reduction of AMP and GMP production, the accumulated intermediates of PDNS might also affect cellular homeostasis. Recently, transcriptome changes were studied in GART, ADSL, and ATIC knockouts in HeLa cells that were genetically edited by the CRISPR-Cas9 technique. It has been shown that transcriptional regulation of these cells is influenced by PDNS deficiencies [23,24,25]. Performing genome editing in low-passage primary human cells is notoriously difficult, and experiments conducted in fibroblasts of PDNS-deficient patients failed to detect biochemical abnormalities, even though sensitive LC-MS/MS methodology was used [20]. PDNS-deficient cell lines must maintain proliferation under purine-free conditions, as the formation of functional purinosome requires 2 h in purine-free medium to form in HeLa cells and is rapidly dissolved when purines are added back into the growth medium [2].

To identify the intermediates of PDNS accumulating in CRISPR-Cas9 genome-edited HeLa cells deficient in the individual steps of PDNS, liquid chromatography coupled with a high-resolution mass spectrometry (LC-HRMS) method was developed in our previous study [26]. In the current study, we applied the method to gain a general overview of cellular metabolism affected by different knockouts of PDNS enzymes representing both the known and the potential disorders of this pathway as well as to confirm the accumulation of PDNS intermediates and potentially identify other statistically significant molecules. Targeted metabolomic analysis was focused on the impact of the defects on central metabolic pathways and understanding the pathobiochemistry. The experiment was conducted under purine-free cultivation conditions since it is necessary for purinosome formation and thus PDNS activity [6]. It has been shown that transcriptional regulation of HeLa cells deficient in PDNS is influenced by PDNS deficiencies [23,24,25]. These transcriptomics analyses found differences in cellular processes involving muscle function, nervous system development, embryonic development, etc. Of the metabolic processes, only glycosaminoglycan synthesis and arachidonic acid metabolism were altered. Macromolecular metabolites affected by these processes are outside the scope of both the analytical approaches applied in our study.

## 2. Results

### 2.1. Untargeted Metabolomic Analysis

The Compound Discoverer software detected 11,729 features in all studied groups. The features were defined by molecular weight (*m*/*z* range 70–1500) and retention time (1.5–17.0 min). To reduce the number of features detected, several filters were applied in Compound Discoverer (see Table S1 in Supplementary data). Subsequently, correlation-based reduction of feature multiplicities (CROP) was performed on the data to remove fragments and adducts [27]. After data preprocessing (included in the Metabol package), the final number of features was 739.

**Figure 1:**
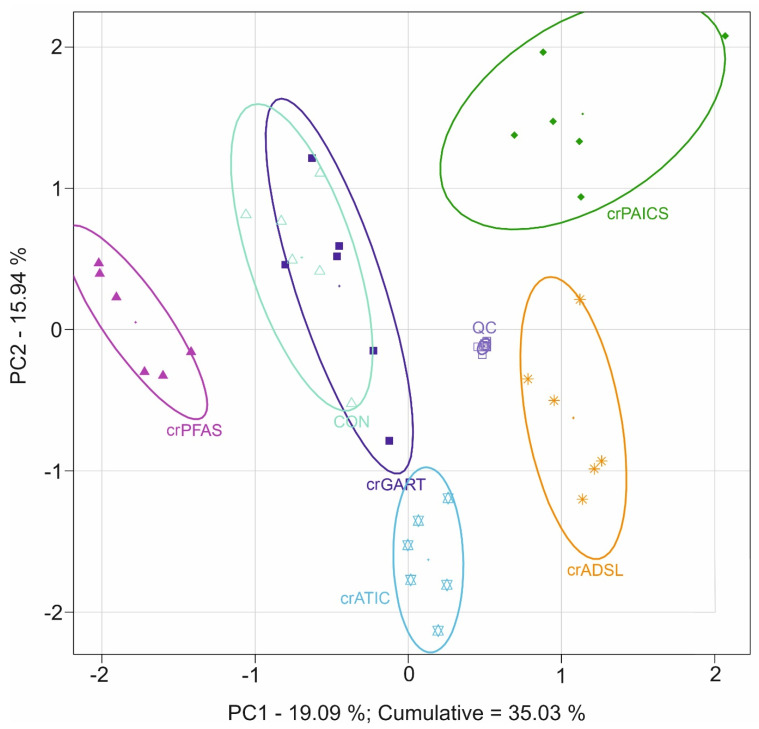
Principal component analysis of HeLa deficient and control cells that were subjected to an untargeted metabolomic analysis. Full line ellipses represent a 75% confidence interval. Congruence of crGART cells with control cells is caused by the lack of accumulation of primary metabolite in the pathway due to chemical instability; see Section 4.1 Untargeted metabolomics. Symbols: crGART cells (dark blue ■), crPFAS cells (purple ▲), crPAICS cells (dark green ♦), crADSL cells (orange ∗), crATIC cells (light blue ✡), control cells (light green △), QC samples (grey □).

**Figure 2:**
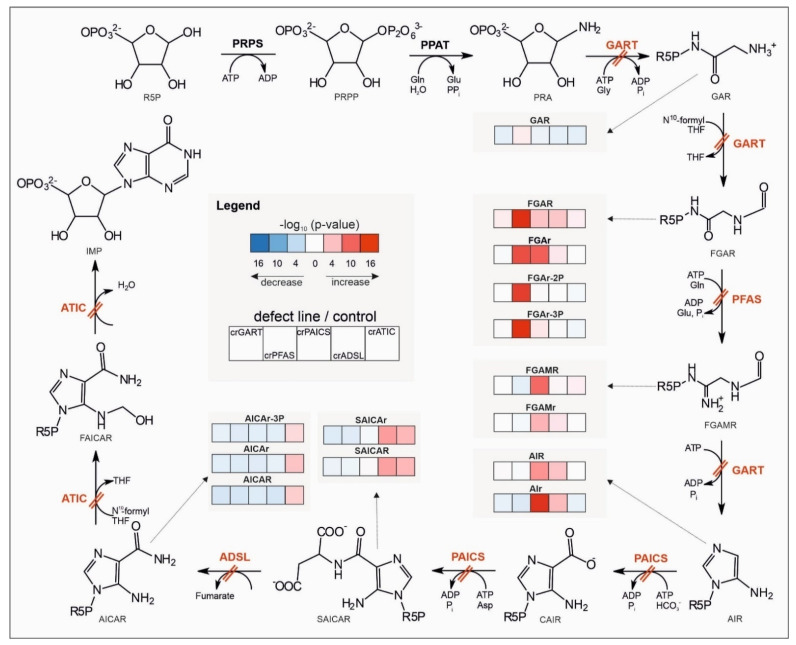
Accumulation of intermediates and their de-/phosphorylated forms along the PDNS pathway due to individual defects of enzymes. Enzyme defects are shown by a double red line across a particular reaction of the pathway. For a given metabolite, the blue/red color of a box represents decreased/increased level of the deficient cell line compared to controls according to ln (fold change), while the shade reflects −log10 (*p*-value) with a lighter/darker color for a statistically less/more significant difference (based on a t-test). Abbreviations: phosphoribosylamine (PRA), glycinamideribonucleotide (GAR), N-formylglycinamide ribonucleotide (FGAR), diphosphate form of FGAR (FGAr-2P), triphosphate form of FGAR (FGAr-3P), N-formylglycinamidine ribonucleotide (FGAMR), aminoimidazole ribonucleotide (AIR), carboxyaminoimidazole ribonucleotide (CAIR), N-succinocarboxamide-5-aminoimidazole ribonucleotide (SAICAR), 5-aminoimidazole-4-carboxamide ribonucleotide (AICAR), triphosphate form of AICAR (AICAr-3P), 5-formamido-4-imidazolecarboxamide ribonucleotide (FAICAR), inosine-5-monophosphate (IMP), glutamine (Gln), glutamate (Glu), pyrophosphate (PPi), adenosine-5-triphosphate (ATP), adenosine-5-diphosphate (ADP), glycine (Gly), phosphate (Pi), *N*^10^-formyl tetrahydrofolate (*N*^10^-formyl THF), tetrahydrofolate (THF), hydrogen carbonic acid (HCO^3−^), aspartate (Asp). Riboside forms of PDNS intermediates are marked with small letter r. Enzyme abbreviations are described in the second paragraph of the Introduction.

The 2D PCA score plot shows a clear separation of crPFAS, crPAICS, crADSL, and crATIC cells from controls with a cumulative explained variability of 35.03% (Figure 1). CrPFAS, crPAICS, crADSL, and crATIC cells separated from controls, whereas crGART cells clustered with controls. The 3D PCA score plots are shown in Figure S2 in Supplementary data. The accumulation of PDNS intermediates due to individual defects is shown in Figure 2.

For every deficient cell line (compared to control cells), the first twenty most discriminating features from orthogonal partial least squares-discriminant analysis variable importance in projection (OPLS-DA VIP ) score plots were subjected to fragmentation analysis in order of their structural identification. Generally, the most discriminating features in all deficient cell lines were identified as PDNS intermediates, their dephosphorylated analogs, and occasionally their di- or triphosphate analogs (Tables S2–S6 in Supplementary data). Three lipids were identified at Metabolomics Standards Initiative (MSI) level 2—two phosphocholines and one lipid belonging to phosphatidylserines. One phosphatidylcholine was identified at MSI level 3. The majority of lipids were found to be decreased in deficient cells compared to controls (Figure S3 in Supplementary data).

### 2.2. Targeted Metabolomic Analysis

**Figure 3:**
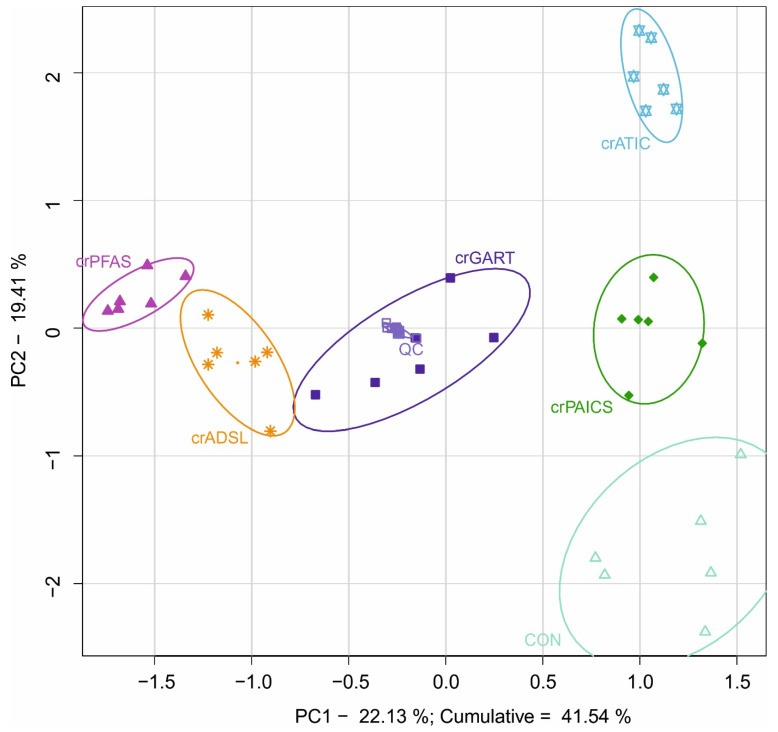
Principal component analysis of HeLa deficient and control cells that were subjected to targeted metabolomic analysis. Full line ellipses represent a 75% confidence interval. All PDNS-deficient cell lines are separated from each other and concurrently from the control cells due to the differences in metabolic profiles other than related directly to PDNS (see Section 4.2 Targeted metabolomics). To elucidate metabolic changes outside the PDNS pathway, intermediary PDNS ribotides and their direct metabolic products were excluded from the analysis. Symbols: crGART cells (dark blue ■), crPFAS cells (purple ▲), crPAICS cells (dark green ♦), crADSL cells (orange ∗), crATIC cells (light blue ✡), control cells (light green △), QC samples (grey □).

Using targeted metabolomic analysis, a total of 183 metabolites were determined by LC-MS/MS analysis of HeLa deficient and control cells. The 2D PCA score plot model with a cumulative explained variability of 41.54% shows a clear separation of deficient and control cells based on differences in metabolic profiles (Figure 3). The same separation of groups is seen also in 3D PCA score plot (Figure S4 in Supplementary data).

**Figure 4:**
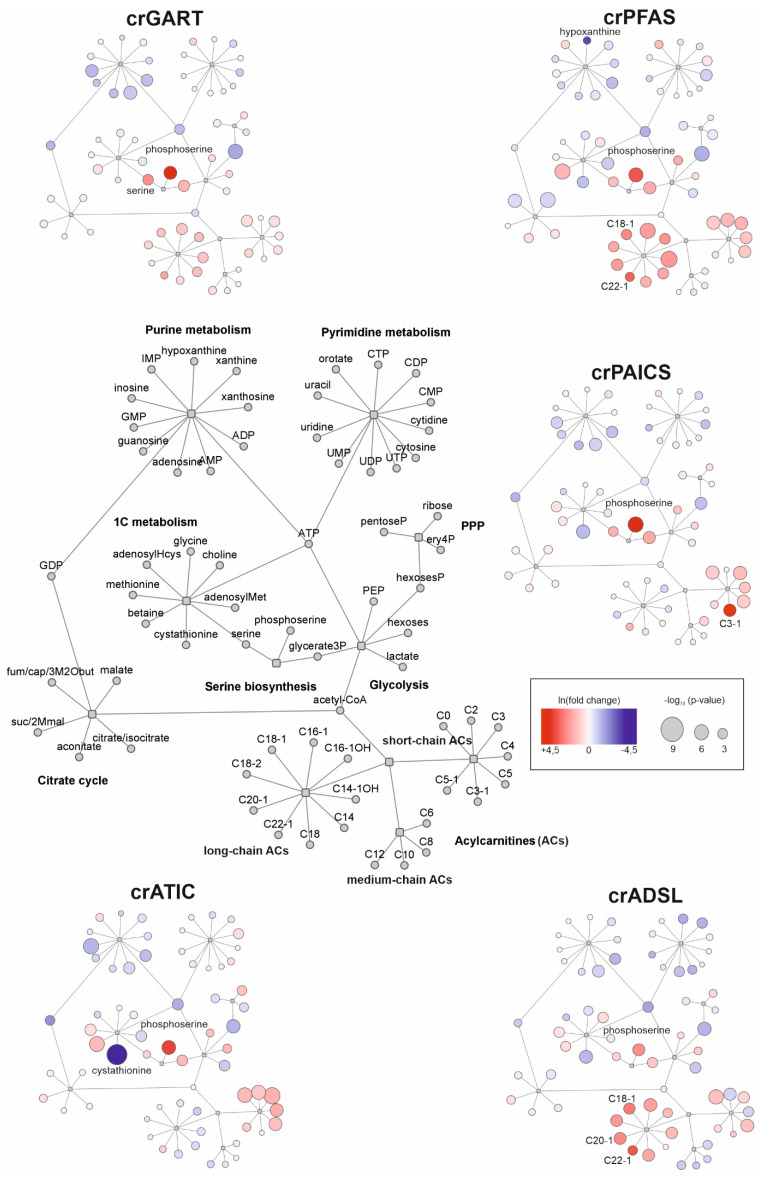
Visualization of the changes detected in cellular metabolism of PDNS-deficient cells compared to control cells performed in Cytoscape software. The general metabolite network of affected pathways is placed central left. Shades of blue/red show decreased/increased metabolites of the deficient cells compared to control cells (based on ln (fold change)), respectively. Colors refer to the natural logarithm of fold change. The size of the dots is given by −log_10_ (*p*-value). Metabolites exhibiting fold change of 3 or more (in absolute values) are named in the picture. The list of metabolite abbreviations is in Table S12 in Supplementary Data. Squares represent parent metabolite interconnection into metabolic subspace, e.g., central ATP represented in the center of purine metabolism.

OPLS-DA VIP score plots were constructed to find the most discriminating metabolites in each pair of deficient and control cells. Then, metabolites with a VIP score higher than one were analyzed in Cytoscape software to further reduce the number of statistically significant metabolites, and mainly, to identify metabolic pathways with the most statistically significant differences (based on VIP score) seen between control and deficient cells (Tables S7–S11 of Supplementary Data). Visualization of the most affected metabolic pathways of the individual defects is depicted in Figure 4. The color transition from red to blue is determined by the difference of medians in clr coefficients between PDNS-deficient cell lines and the control group. Dot size represents statistical significance (given by −log_10_ (*p*-value)).

The energy status of all cell lines was calculated as an adenylate energy charge. It was detected to be lower in crPFAS, crADSL, and crATIC deficient cells compared to crPAICS and control cells (Figure S5 in Supplementary data).

## 3. Discussion

In the present study, we used both untargeted and targeted metabolomics to obtain a global view of the changes in the metabolome of HeLa cells induced by individual defects in PDNS. Effects of accumulation of pathological substrates are not limited to affecting biological equilibria. However, other features such as signaling can drive the overall cellular response. The study is restrained by its static nature, and the results of the experiment point to the necessity of kinetic experimental design to shed more light on the underlying mechanisms and their regulation. Despite its limitations, PDNS-deficient HeLa cells represent the closest available model of PDNS metabolic disorders to humans, and this study could help in unraveling the nature of these still not well-understood enzyme defects. The metabolic behavior of cells with disrupted PDNS might also help in studies focusing on therapeutic targets for the treatment of cancers with a high purine demand.

### 3.1. Untargeted Metabolomics

Untargeted metabolomic analysis of crGART, crPFAS, crPAICS, crADSL, and crATIC cell lines was performed to gain a general view of the metabolic response of the cells outside of the scope of the targeted analysis. We analyzed the accumulation of PDNS intermediates and their dephosphorylated analogs employing fragmentation (based on exact mass fragmentation, and structural prediction of chromatographic behavior), and all other statistically significant metabolites that were not covered by a targeted analysis (based on their VIP score). Multivariate statistical analysis revealed that the build-up of metabolites of PDNS had a major impact on separating defective cell lines and controls. Then, we performed a targeted metabolomic analysis to examine major changes in fundamental metabolic pathways (see Section 4.2 Targeted analysis).

An unsupervised PCA model shows a clear separation of crPFAS, crPAICS, crADSL, and crATIC cell lines from control cells, whereas crGART cells overlap with control cells (Figure 1). Except for crGART, all other deficient cell lines showed accumulation of a substrate of an affected enzymatic reaction (Figure 2). CrGART cell line did not accumulate PRA due to its instability (i.e., a decomposition of chemically unstable PRA to PRPP) in vivo [28], as was also shown in our previous studies [26,29]. No other metabolic changes downstream of the pathway were detected in crGART cells since they carry a mutated form of the trifunctional enzyme GART, which is not expressed at all. We observed a slight separation of crGART and control cells in 3D PCA score plots (Figure S2 in Supplementary data). However, we were not able to find a connection to the metabolites of the affected PDNS pathway. Individual pairs of deficient cell lines compared to controls are shown in Figures S6–S10 of Supplementary data. Arrows constructed from loadings representing PDNS metabolites point in the direction of the deficient cell line. It implies that PDNS metabolites had a major impact on separation in 2D PCA for all cell lines (separate pairs of deficient cell line and control cells), as also supported by the high cumulative explained variability (about 70% in all cases, See Figures S6–S10 in Supplementary data). Accumulation of PDNS metabolites upstream of the enzymatic block was also detected as described previously [15,26,30,31], suggesting a shift in the equilibria of enzymatic reactions along the metabolic pathway (Figure 2). We also detected the accumulated mono- and triphosphate forms of AICAr in the crATIC cell line, which is in agreement with the analysis of erythrocytes of a patient suffering from AICA-ribosiduria [16]. Unlike adenine nucleotides, which are synthesized by nucleoside mono-/diphosphate kinases, AICAr-3P is synthesized directly from AICAR by phosphoribosyl pyrophosphate synthetase (PRPS, EC 2.7.6.1) action. Using PRPS is not a predominant route for adenine nucleotides, and this might be the cause of different ratios between those two types of nucleotides (ratios of AICAR/AICAr-2P/AICAr-3P and AMP/ADP/ATP) [32]. It was proposed that the diphosphate form of AICAr (AICAr-2P) is likely a product of the intracellular degradation of AICAr-3P or originates during metabolite extraction, rather than being an intermediate of AICAr-3P synthesis [33]. Moreover, we detected FGAr-2P and FGAr-3P in crPFAS cell line, where we expected an analogous mechanism of origin. Except for AICAr, no other di- and triphosphate analogs of PDNS metabolites have been reported in living cells so far. Statistical significance of the intermediates of the PDNS pathway was the highest (*p*-values 10 × 10^−7^–10 × 10^−15^), whereas metabolites involved in other metabolic pathways were changed with *p*-values of up to 10 × 10^−7^. This is in agreement with the picture we typically see in monogenic enzyme defects.

### 3.2. Targeted Metabolomics

Metabolites detected by a targeted analysis were statistically analyzed, and visualization of the major changes in cellular metabolism of PDNS-deficient cells compared to control cells (Figure 4) was performed in Cytoscape. To elucidate metabolic changes outside the PDNS pathway, intermediary PDNS ribotides and their direct metabolic products were excluded from the analysis. The most statistically significant changes (based on VIP score of metabolites) were seen in the metabolism of nucleotides, saccharides, acylcarnitines, and one-carbon units (1C), which are all discussed below.

#### 3.2.1. Central Energy Nucleotide Metabolism

In a normal environment with a sufficient supply of external purines, human cells prefer recycling available purines rather than producing them by energy-demanding PDNS [34]. Under given experimental conditions in a purine-free cultivating medium (24 h before harvesting), the cells are forced to utilize this pathway and thus reveal the consequences of defects in PDNS. All deficient cell lines shared decreased levels of adenine and guanine metabolites due to individual enzyme blocks along PDNS combined with cultivation of cells in purine starvation conditions. Earlier studies of ADSL-deficient patients have shown that the residual activity of ADSL (3–40%) is sufficient to keep the flux through PDNS unaffected [18,35]. Enzyme activity assays of the cell lines used in our study revealed detectable enzyme activity only in crADSL cells (2.6%) [29].

Adenylate energy charge (AEC), calculated as (ATP + ½ ADP)/(ATP + ADP + AMP), measures the energy status of the cell [36]. The cells fluctuate within narrow limits and with increasing AEC, the supply of ATP and other important biosynthetic intermediates is suspended by inhibition of, e.g., phosphofructokinase and pyruvate kinase. Contrarily, ATP-demanding pathways are activated due to the activation of citrate lyase (lipogenesis) and PRPS (nucleotide synthesis) [36,37]. The AEC values are maintained despite large fluctuations in the concentrations of adenosine nucleotides (compare Figures S5 and S11 in Supplementary data). It has been shown that the adenylate pool may drop to about 30% of its normal value before the AEC is measurably affected [38,39]. Since the AEC of all deficient cell lines remained within the physiological range throughout the experiment (Figure S5 in Supplementary data) and AMP, ADP, and ATP (Figure S11 in Supplementary data) concentrations were reduced in all deficient cell lines over control cells, there could be reduced availability of ATP for biochemical interconversions utilizing ATP. In this case, it could be attributed to cultivation conditions where the defect in the purine pathway is combined with auxotrophic conditions. This effect could be the underlying mechanism of changes observed in the biosynthesis of pyrimidines, where the decreased level of ATP probably caused a decreased level of pyrimidine nucleotides (e.g., cytidine di/triphosphate) compared to control cells since the biosynthesis of pyrimidines also requires high consumption of ATP. Furthermore, low levels of ribose-5-phosphate (a precursor of PRPP), as well as glucose-6-phosphate, observed in deficient cell lines, could cause slower pyrimidine biosynthesis.

#### 3.2.2. Carbohydrate Metabolism

Decreased levels of phosphohexoses and increased levels of glucose were seen in all deficient cell lines. This intracellular imbalance might be attributed to lower concentrations of ATP in the deficient cells (see Section 3.2.1) and direct consumption of ATP in the hexokinase (EC 2.7.1.1) reactions involved. No hyperglycemia was reported in ADSL-deficient patients, and only transient hypoglycemia was described in ATIC-deficient patients [15,30] that was associated with increased activation of the insulin pathway in hepatocytes [15,40]. We observed elevated levels of glycolytic intermediates glycerate-3-phosphate and phosphoenolpyruvate in the defective cell lines. It was reported for several tumor cells that the glycolytic flux can be diverted toward serine synthesis from the glycolytic intermediate 3-phosphoglycerate [41,42,43]. The first and rate-limiting enzyme of the serine synthesis pathway (SSP), phosphoglycerate dehydrogenase (EC 1.1.11.95), is inhibited by an increased level of serine [44] which most probably accumulates due to the inability of PDNS (and other biosynthetic pathways) to consume 1C units generated from this amino acid (see more in Section 3.2.4 One-carbon metabolism). Decreased levels of intracellular lactate were detected in the deficient cell lines compared to controls. A study of the effects of AICAR on the metabolism of HUVEC cells using nuclear magnetic resonance showed a 3-fold decrease in lactate because these cells prefer fatty acid oxidation over glycolysis [45], which is in agreement with our data in crATIC cells. However, changes of lactate levels in patients suffering from AICA-ribosiduria were not reported [15,16].

#### 3.2.3. Metabolism of Acylcarnitines

Acylcarnitines (ACs) are a large group of esters of L-carnitine and fatty acids comprising more than 1200 members that participate in sugar and lipid metabolism, metabolism of branched-chain amino acids (BCAA), production of ketone bodies, oxidation of fatty acids, and other cellular processes [46]. Short-, medium-, and long-chain acylcarnitines showed the greatest mutual variability among the individual deficient cell lines compared to other metabolites (Figure 4). It points towards defective enzyme-specific changes (induced by accumulating PDNS intermediates) more than global PDNS pathway-related changes. In all deficient cells except for crADSL, we observed an increased level of intracellular short-chain ACs, while medium-chain ACs appeared to be minimally affected. In crADSL cells, we observed changes in even short-chain ACs. In crADSL, all mitochondrial β-oxidation intermediates (C2–C12) were decreased compared to controls. Tai et al. [47] showed an elevation in some metabolites reflective of BCAA catabolism (e.g., glutamate, alanine, C3, and C5) when studying insulin resistance in obese individuals [47]. We did not observe a decrease in the levels of BCAA, but the levels of glutamate and alanine were increased. AICAR accumulating in crATIC cells may be responsible for the elevation of short-chain ACs itself by accelerating β-oxidation of fatty acids [48]. By activating AMPK, it triggers the inactivation of acetyl-CoA carboxylase, thus decreasing the level of malonyl-CoA, which is an inhibitor of carnitine palmitoyltransferase 1 (CPT1, EC 2.3.1.21). Activation of CPT1 follows, allowing a higher rate of fatty acid oxidation. Increased fatty acid oxidation due to AMPK activation by AICAR was shown in HUVEC cells [45]. CrGART, crPFAS, and crADSL cells exhibited a considerable increase in even long-chain ACs (both saturated and unsaturated), pointing to an incomplete β-oxidation of fatty acids, as was shown in the study of insulin resistance in murine myocytes. In this experiment, a large proportion of fatty acids entering the mitochondria were only partially degraded, and an increase in even long-chain ACs (C6–C22) was detected [49]. Metabolomic profiling of patients with ADSL deficiency did not reveal any diagnostic acylcarnitine in plasma samples [50].

#### 3.2.4. One-Carbon Metabolism

Increased levels of serine and intermediates of its biosynthesis (glycerate-3P, phosphoserine) were observed in all deficient cell lines. Serine transmethylation product (catalyzed by serine hydroxymethyltransferase; SHMT, EC 2.1.2.1), glycine, was decreased in crGART, crPFAS, and crADSL cells. Subedi et al. reported higher basal levels of SSP enzymes in HeLa cells [51]. A study of the NCI60 panel showed that glycine conversion increases the rate of proliferation and significantly supplies PDNS [42], which were both metabolic processes greatly affected in our study given the cellular model and experimental conditions. Serine is supposed to be a major donor into 1C metabolism based on an isotope tracer analysis, and serine-derived units are preferentially used for nucleotide synthesis [52]. The observed changes in the serine–glycine ratio can be attributed to the low utilization of 1C units in the PDNS pathway.

Decreased levels of S-adenosylhomocysteine and S-adenosylmethionine and increased levels of methionine seen in all deficient cell lines could also be attributed to the lower level of ATP (discussed in Section 3.2.1 Central energy nucleotide metabolism) affecting S-adenosylmethionine synthase (EC 2.5.1.6) reaction. Considering the role of S-adenosylmethionine as a crucial methyl donor, many methylation processes such as the conversion of lysine, arginine, histone, DNA, and RNA might be affected [53,54]. A significant decrease in the level of cystathionine (a part of the transsulfuration pathway) was observed in all deficient cell lines, most profoundly in crATIC cells. This condition might be induced by low levels of SAM and adenosylHcys, which both serve as allosteric activators of cystathionine-beta-synthase (EC 4.2.1.22)—an enzyme catalyzing the synthesis of cystathionine from serine and homocysteine. Concerning crATIC cells, accumulated AICAR alone could potentially be responsible for changes in 1C metabolism. AICAR, together with its triphosphate analog, AICAr-3P, was found to regulate the expression of 1C metabolism genes in many bacteria through riboswitch activation [55]. It has been hypothesized that AICAR may also be a major regulator of 1C metabolism in eukaryotes considering the conservation of this pathway throughout all domains of life [56].

## 4. Materials and Methods

### 4.1. Chemicals

Water, methanol, and acetonitrile, all LC-MS grade, were purchased from Sigma-Aldrich (St. Louis, MO, USA). Ammonium hydroxide and acetic acid were also purchased from Sigma-Aldrich. Minimum Essential Medium (MEM) was obtained from BioSera (Nuaille, France). Calf intestinal alkaline phosphatase (CIP) and NEB3 buffer were purchased from New England Biolabs (NEB, Ipswich, MA, USA), and Dulbecco’s minimum essential medium (DMEM), F12 nutrient mix, and fetal bovine serum (FBS) were obtained from Life Technologies, ThermoFisher Scientific (Waltham, MA, USA).

### 4.2. Cell Cultivation, Harvesting, and Sample Preparation

CRISPR-Cas9 genome-edited HeLa cells deficient in GART, PFAS, PAICS, ADSL, and ATIC enzymes (crGART, crPFAS, crPAICS, crADSL, and crATIC cells), prepared by Baresova et al. [29], were used in this study. The HeLa cell line used as control was the same as that used for genome edition. HeLa cells used as a control were not edited by CRISPR-Cas9 technique. The cells were grown in DMEM/F12 nutrient mix medium supplemented with 10% FBS and 1% penicillin/streptomycin. The medium of knockout cells was enriched with 3 × 10^−5^ M adenine. Twenty-four hours before harvesting, the cells were transferred to purine-depleted DMEM supplemented with dialyzed 10% FBS and 1% penicillin/streptomycin. The cells were grown in 25-cm^2^ flasks under standard culture conditions (humidified atmosphere, 5% CO_2_, 37 °C) until 80–90% confluence (approx. 1 million cells). Cell lines were cultivated in hexaplicate (seeded into six separate cultivation flasks for each PDNS-deficient cell line and cultivated until the experiment).

The quenching method, described by Wojtowicz et al. [57], was used for cell harvesting with modified volumes of washing and extraction methanol. After removal of the culture medium, the cells were quenched by spraying out 20 mL 60% aqueous cold methanol (*v*/*v*, −50 °C) using a plastic syringe with a bent needle. The flasks were kept on ice, and 1 mL of 80% aqueous cold methanol (*v*/*v*, −50 °C) was added for metabolite extraction. A cell scraper was used for mechanical detachment. Cell debris in methanol was drained out by a pipette and another 2 mL of an extraction solution was added for washing the flask. Both methanol extracts were combined, sonicated (30 s), and centrifuged (1800× *g*, 5 min, 4 °C). Supernatants were freeze-dried. Each lyophilizate was mixed with 200 μL of 80% cold methanol and centrifuged (15,000× *g*, 15 min, 4 °C). Supernatants were transferred and divided into two vials for subsequent analyses. Given the technical performance of cell harvesting (speed needed for an immediate quenching of cellular metabolism; cell scraping profoundly affecting cell integrity), cell count was not available from the experimental flask, and we calculated metabolite concentrations using centered log-ratio transformation (see Section 4.6 Statistical analysis).

### 4.3. Untargeted Metabolomic Analysis

All samples for the untargeted analysis were analyzed in one batch in a randomized order. Quality control (QC) samples were prepared by pooling all extracts of deficient cell lines and control samples (10 μL), and they were used for the first ten injections to stabilize the system and afterward as every fifth injection. LC-HRMS analysis was performed on an Ultimate 3000 Rapid Separation system (Dionex, Sunnyvale, CA, USA) coupled to an Orbitrap Elite mass spectrometer (Thermo Fischer Scientific, Waltham, MA, USA). The LC-HRMS system was controlled by Chromeleon Xpress 6.80, Dionex DCMSLink 2.12, Thermo Tune Plus 2.7.0.1103 SP1 (Thermo Fischer Scientific), and Xcalibur 2.2 SP1 software (Thermo Fischer Scientific). The stationary phase employed an aqueous normal phase separation system using aminopropyl column Luna NH_2_ 3 μM 100 Å, 100 × 2 mm (Phenomenex, Torrance, CA, USA). Chromatographic separation was performed at 35 °C. Binary gradient elution consisted of 20 mM ammonium acetate in water, pH 9.75 (mobile phase A), and acetonitrile (mobile phase B). The gradient elution at a flow rate of 0.3 mL/min was as follows: t = 0.0, 95% B; t = 7.0–13.0, 10% B; t = 14.0–17.0, 95% B. The injection volume was 5 μL. The temperature of an autosampler was held constant at 4 °C.

The Orbitrap Elite mass spectrometer operated in positive full-scan mode at a resolution of 60,000 full width at half maximum (FWHM) within a mass to charge ratio (*m*/*z*) range of 70–1500. The setting of the electrospray ionization was: heater temperature of 300 °C, a capillary temperature of 350 °C, sheath gas of 35 arb. units, auxiliary gas of 10 arb. units and the source voltage of +3.0 kV. The exact mass accuracy was below 5 ppm. Data were acquired in a profile mode.

The most discriminant features from OPLS-DA VIP (see more in Section 4.6 Statistical analysis) underwent a fragmentation analysis to identify their structure. Fragmentation spectra of specific *m*/*z* were acquired on Orbitrap Elite using the collision-induced dissociation (CID) fragmentation method. Settings for MS2 were: activation Q of 0.25, activation time of 10 ms, normalized collision energy of 35%, and the resolution of 15,000. The analysis was operated in both positive and negative modes. Chromatographic separation and ion source settings were identical to the untargeted analysis. Metabolomics Standards Initiative (MSI) rules were followed to describe the level of identification. For MSI level 1 (confidently identified compounds), *m*/*z*, fragmentation spectra, and retention time were compared to available standards. The comparison of exact mass and fragmentation spectra with databases (mzCloud, Metlin, LipidMaps) was performed for MSI level 2 (putatively annotated compounds). For MSI level 3 (putatively annotated compound classes), the main class of a compound was determined based on the exact mass, specific fragmentation of exact masses, and region of elution from the chromatographic column. For MSI level 4 (unknown compounds), the fragmentation spectra could not be compared because they were not successfully matched in the above-mentioned databases [58].

### 4.4. Targeted Metabolomic Analysis

Targeted analysis was performed by liquid chromatography coupled with tandem mass spectrometry (LC-MS/MS) using the Ultimate 3000 Rapid Separation system (Dionex, Sunnyvale, CA, USA) coupled to triple-quadrupole mass spectrometer Triple Quad 6500 (SCIEX, Framingham, MA, USA). The parameters for LC separation were the same as in the untargeted analysis. The mass spectrometer operated in polarity switching mode using scheduled multiple reaction monitoring (MRM). Both quadrupoles were set at unit resolution. The parameters of the ion source and gases were as follows: ion spray voltage of +5500 V and –4500 V, source temperature 400 °C, curtain gas of 40 psi, and both ion source gases of 40 psi. Declustering potential, collision energy, entrance potential, and collision cell exit potential were previously optimized using authentic chemical standards [59]. Metabolites changed in untargeted analysis that were related directly to PDNS pathway were excluded from the targeted data analysis (see Section 4.2). The instrument was controlled by the Analyst 1.6.2 software (AB SCIEX, Foster City, CA, USA).

### 4.5. Data Processing

The raw data files from the untargeted metabolomic analysis were processed in the Compound Discoverer^TM^ 3.0.0.294 software (Thermo Scientific, Fremont, CA, USA). The processing workflow employed retention time alignment, peak picking, adduct merging, gap-filling, background subtraction, as well as identification nodes such as composition prediction and multiple compound databases and spectral library search. The detailed parameters are shown in Figure S1 of Supplementary data. Correlation-based reduction of feature multiplicities (CROP) was performed to merge in-source fragments and other multiplicities in the data (correlation coefficient threshold of 0.95, RT window 0.06 min) [27]. Data acquired in the targeted metabolomics analysis were processed manually in the MultiQuant 3.0 software (AB SCIEX, Foster City, CA, USA).

### 4.6. Statistical Analysis

Data from both untargeted and targeted analyses were evaluated in statistical software R (version 3.5.0, www.r-project.org, accessed on 30 November 2019) using our Metabol package (https://github.com/AlzbetaG/Metabol, accessed on 30 November 2019). The quality control-based LOESS signal correction [60,61] was applied to the data. Based on the QC samples, coefficients of variation (CV) were calculated and features/metabolites with a CV value higher than 30% were rejected from further processing. Centered log-ratio transformation was applied to the data structure [62] to adjust for “size effect” as a substitute to normalization to protein counts. Centered log-ratio coefficients express the measured data as logarithmized dominance of the metabolites to the average behavior of the whole metabolome. This representation of a so-called log-ratio approach allows revealing the relative information hidden in the data structure. Both univariate and multivariate statistical analyses were used for data evaluation and visualization. Using Bonferroni correction (α = 0.05/number of features), the *p*-values were calculated by multiple t-testing. Unsupervised principal component analysis (PCA) and supervised orthogonal partial least squares-discriminant analysis (OPLS-DA) were applied to the data. OPLS-DA variable importance in projection (VIP) score plots were used for the determination of the most discriminant features/metabolites. In the untargeted analysis, the first 20 features according to the highest score from OPLS-DA VIP plots were chosen for the subsequent fragmentation analysis. Metabolites acquired from the targeted analysis were processed in Cytoscape (version 3.8.2, https://cytoscape.org/, accessed on 5 June 2021) to visualize the metabolic pathways contributing the most to the separation of individual groups of deficient and control cells.

## 5. Conclusions

Many metabolic patterns were generally shared by all deficient cell lines compared to controls. The most statistically significant metabolites differentiating PDNS-deficient cells from controls were the substrates of the defective enzymes. Disturbed PDNS resulted in an altered pool of adenine and guanine nucleotides, yet the adenylate energy charge was not different from controls. In all PDNS-deficient cell lines, we observed changes in remethylation cycle metabolites that implicate lower methylation activity of the deficient cells that might alter many processes including modification states of proteins, DNA and RNA. Diversity in the levels of acylcarnitine observed in the individual deficient cell lines might be associated with a specific enzyme deficiency rather than global changes related to the PDNS pathway. Although the translation of cellular in vitro models to patient metabolism is very difficult and far from straightforward, this study could be beneficial in unraveling the nature of these still not well-understood enzyme defects. Current findings are cell line-specific, and additional studies on other CRISPR-Cas9 edited cell lines will be needed to draw general conclusions once these cellular models are available.

## Data Availability

The data presented in this study are available in article and Supplementary Material.

## Supplementary Materials

The following supporting information can be downloaded at: https://www.mdpi.com/article/10.3390/metabo12030241/s1, Figure S1: Compound Discoverer workflow parameters; Figure S2: 3D Principal component analysis of HeLa deficient and control cells that were subjected to an untargeted metabolomic analysis; Figure S3: Boxplots of significant lipids identified in all cell lines; Figure S4: 3D Principal component analysis of HeLa deficient and control cells that were subjected to a targeted metabolomic analysis; Figure S5: The adenylate energy charge plot shows a comparison of box plots of deficient cell lines and controls; Figure S6: PCA biplots of GART-deficient cells compared to control group subjected to an untargeted analysis; Figure S7: PCA biplots of PFAS-deficient cells compared to control group subjected to an untargeted analysis; Figure S8: PCA biplots of PAICS-deficient cells compared to control group subjected to an untargeted analysis; Figure S9: PCA biplots of ADSL-deficient cells compared to control group subjected to an untargeted analysis; Figure S10: PCA biplots of ATIC-deficient cells compared to control group subjected to an untargeted analysis; Figure S11: Boxplots of adenosine nucleotides in PDNS deficient cell lines and control cells, Table S1: Filters used in CD for feature number reduction; Table S2: The top 20 most significant features of VIP-OPLS-DA detected in crGART cells compared to controls in untargeted analysis; Table S3: The top 20 most significant features of VIP-OPLS-DA detected in crPFAS cells compared to controls in untargeted analysis; Table S4: The top 20 most significant features of VIP-OPLS-DA detected in crPAICS cells compared to controls in untargeted analysis; Table S5: The top 20 most significant features of VIP-OPLS-DA detected in crADSL cells compared to controls in untargeted analysis; Table S6: The top 20 most significant features of VIP-OPLS-DA detected in crATIC cells compared to controls in untargeted analysis; Table S7: Significant metabolites of VIP-OPLS-DA (mean VIP higher than 1) detected in crGART cells compared to control in targeted analysis; Table S8: Significant metabolites of VIP-OPLS-DA (mean VIP higher than 1) detected in crPFAS cells compared to control in targeted analysis; Table S9: Significant metabolites of VIP-OPLS-DA (mean VIP higher than 1) detected in crPAICS cells compared to control in targeted analysis; Table S10: Significant metabolites of VIP-OPLS-DA (mean VIP higher than 1) detected in crADSL cells compared to control in targeted analysis; Table S11: Significant metabolites of VIP-OPLS-DA (mean VIP higher than 1) detected in crATIC cells compared to control in targeted analysis; Table S12: List of abbreviations.

## Abbreviations

Adenosine-5-triphosphate, (ATP); adenylate energy charge, (AEC); adenylosuccinate lyase, (ADSL); amidophosphoribosyltransferase, (PPAT); AMP-activated kinase, (AMPK); collision-induced dissociation, (CID); correlation-based reduction of feature multiplicities, (CROP); full width at half maximum, (FWHM); high-resolution mass spectrometry, (LC-HRMS); inosine-5-monophosphate, (IMP); ion beam mass spectrometry, (GCIB-SIMS); orthogonal partial least squares-discriminant analysis, (OPLS-DA); phosphoribosylaminoimidazole carboxylase and phosphoribosylaminoimidazolesuccinocarboxamide synthase, (PAICS); phosphoribosylaminoimidazole synthetase and phosphoribosylformylglycinamidine synthase, (PFAS); phosphoribosylaminoimidazolecarboxamide formyltransferase and IMP cyclohydrolase, (ATIC); phosphoribosylglycinamide synthetase and phosphoribosylglycinamide formyltransferase and phosphoribosylaminoimidazole synthetase, (GART); principal component analysis, (PCA); purine de novo synthesis, (PDNS); S-adenosylhomocysteine, (adenosylHcys); S-adenosylmethionine, (adenosylMet); succinyl aminoimidazole carboxamide riboside, (SAICAr); succinyladenosine, (S-Ado); succinyladenosine monophosphate, (SAMP); variable importance in projection, (VIP); 5-amino-4-imidazolecarboxamide riboside, (AICAr); 5-phosphoribosyl-1-pyrophosphate, (PRPP).

## References

[1] Curto R., Voit E.O., Sorribas A., Cascante M. (1998). Mathematical models of purine metabolism in man. Math. Biosci..

[2] An S., Kumar R., Sheets E.D., Benkovic S.J. (2008). Reversible Compartmentalization of de Novo Purine Biosynthetic Complexes in Living Cells. Science.

[3] Kondo M., Yamaoka T., Honda S., Miwa Y., Katashima R., Moritani M., Yoshimoto K., Hayashi Y., Itakura M. (2000). The Rate of Cell and Growth Is Regulated by Purine Biosynthesis via ATP Production G, to S Phase Transition. J. Biochem..

[4] Deng Y., Gam J., French J.B., Zhao H., An S., Benkovic S.J. (2012). Mapping Protein-Protein Proximity in the Purinosome. J. Biol. Chem..

[5] An S., Deng Y., Tomsho J.W., Kyoung M., Benkovic S.J. (2010). Microtubule-assisted mechanism for functional metabolic macromolecular complex formation. Proc. Natl. Acad. Sci. USA.

[6] French J.B., Jones S.A., Deng H., Pedley A.M., Kim D., Chan C.Y., Hu H., Pugh R.J., Zhao H., Zhang Y. (2016). Spatial colocalization and functional link of purinosomes with mitochondria. Science.

[7] Pedley A.M., Benkovic S.J. (2017). A New View into the Regulation of Purine Metabolism: The Purinosome. Trends Biochem. Sci..

[8] Pareek V., Tian H., Winograd N., Benkovic S.J. (2020). Metabolomics and mass spectrometry imaging reveal channeled de novo purine synthesis in cells. Science.

[9] Yamaoka T., Yano M., Kondo M., Sasaki H., Hino S., Katashima R., Moritani M., Itakura M. (2001). Feedback Inhibition of Amidophosphoribosyltransferase Regulates the Rate of Cell Growth via Purine Nucleotide, DNA, and Protein Syntheses. J. Biol. Chem..

[10] Vergis J.M., Bulock K.G., Fleming K.G., Beardsley G. (2001). Human 5-Aminoimidazole-4-carboxamide Ribonucleotide Transformylase/Inosine 5′-Monophosphate Cyclohydrolase. A bifunctional protein requiring dimerization for transformylase activity but not for cyclohydrolase activity. J. Biol. Chem..

[11] French J.B., Zhao H., An S., Niessen S., Deng Y., Cravatt B.F., Benkovic S.J. (2013). Hsp70/Hsp90 chaperone machinery is involved in the assembly of the purinosome. Proc. Natl. Acad. Sci. USA.

[12] An S., Kyoung M., Allen J.J., Shokat K.M., Benkovic S.J. (2010). Dynamic Regulation of a Metabolic Multi-enzyme Complex by Protein Kinase CK2. J. Biol. Chem..

[13] Wang W., Fridman A., Blackledge W., Connely S., Wilson I.A., Pilz R., Boss G.R. (2009). The Phosphatidylinositol 3-Kinase/Akt Cassette Regulates Purine Nucleotide Synthesis. J. Biol. Chem..

[14] Ben-Sahra I., Hoxhaj G., Ricoult S.J.H., Asara J.M., Manning B.D. (2016). mTORC1 induces purine synthesis through control of the mitochondrial tetrahydrofolate cycle. Science.

[15] Ramond F., Rio M., Héron B., Imbard A., Marie S., Billiemaz K., Denommé-Pichon A., Kuentz P., Ceballos I., Piraud M. (2020). AICA -ribosiduria due to ATIC deficiency: Delineation of the phenotype with three novel cases, and long-term update on the first case. J. Inherit. Metab. Dis..

[16] Marie S., Heron B., Bitoun P., Timmerman T., Van Den Berghe G., Vincent M.-F. (2004). AICA-Ribosiduria: A Novel, Neurologically Devastating Inborn Error of Purine Biosynthesis Caused by Mutation of ATIC. Am. J. Hum. Genet..

[17] Baresova V., Škopová V., Sikora J., Patterson D., Sovova J., Zikanova M., Kmoch S. (2012). Mutations of ATIC and ADSL affect purinosome assembly in cultured skin fibroblasts from patients with AICA-ribosiduria and ADSL deficiency. Hum. Mol. Genet..

[18] Jaeken J., Wadman S.K., Duran M., Van Sprang F.J., Beemer F.A., Holl R.A., Theunissen P.M., De Cock P., van den Bergh F., Vincent M.F. (1988). Adenylosuccinase deficiency: An inborn error of purine nucleotide synthesis. Eur. J. Pediatr..

[19] Jurecka A., Zikanova M., Kmoch S., Tylki-Szymanska A. (2015). Adenylosuccinate lyase deficiency. J. Inherit. Metab. Dis..

[20] Pelet A., Skopova V., Steuerwald U., Baresova V., Zarhrate M., Plaza J.-M., Hnizda A., Krijt M., Souckova O., Wibrand F. (2019). PAICS deficiency, a new defect of of de nvo purine synthesis resulting in multiple congenital anomalies and fatal outcome. Hum. Mol. Genet..

[21] Jurecka A. (2009). Inborn errors of purine and pyrimidine metabolism. J. Inherit. Metab. Dis..

[22] Balasubramaniam S., Duley J.A., Christodoulou J. (2014). Inborn errors of purine metabolism: Clinical update and therapies. J. Inherit. Metab. Dis..

[23] Mazzarino R.C., Baresova V., Zikánová M., Duval N., Wilkinson T.G., Patterson D., Vacano G.N. The CRISPR-Cas9 crGART HeLa transcriptome: A novel cell model of de novo purine synthesis deficiency. BioRxiv.

[24] Mazzarino R.C., Baresova V., Zikánová M., Duval N., Wilkinson T., Patterson D., Vacano G.N. (2019). The CRISPR-Cas9 crADSL HeLa transcriptome: A first step in establishing a model for ADSL deficiency and SAICAR accumulation. Mol. Genet. Metab. Rep..

[25] Mazzarino R.C., Baresova V., Zikánová M., Duval N., Wilkinson T.G., Patterson D., Vacano G.N. (2020). The CRISPR-Cas9 crATIC HeLa transcriptome: Characterization of a novel cellular model of ATIC deficiency and ZMP accumulation. Mol. Genet. Metab. Rep..

[26] Mádrová L., Krijt M., Barešová V., Václavík J., Friedecký D., Dobešová D., Součková O., Škopová V., Adam T., Zikánová M. (2018). Mass spectrometric analysis of purine de novo biosynthesis intermediates. PLoS ONE.

[27] Kouřil Š., de Sousa J., Václavík J., Friedecký D., Adam T. (2020). CROP: Correlation-based reduction of feature multiplicities in untargeted metabolomic data. Bioinformatics.

[28] Schendel F.J., Cheng Y.S., Otvos J.D., Wehrli S., Stubbe J. (1988). Characterization and chemical properties of phosphoribosylamine, an unstable intermediate in the de novo purine biosynthetic pathway. Biochemistry.

[29] Baresova V., Krijt M., Skopova V., Součková O., Kmoch S., Zikanova M. (2016). CRISPR-Cas9 induced mutations along de novo purine synthesis in HeLa cells result in accumulation of individual enzyme substrates and affect purinosome formation. Mol. Genet. Metab..

[30] Jurecka A., Zikanova M., Tylki-Szymanska A., Krijt J., Bogdanska A., Gradowska W., Mullerova K., Sykut-Cegielska J., Kmoch S., Pronicka E. (2008). Clinical, biochemical and molecular findings in seven Polish patients with adenylosuccinate lyase deficiency. Mol. Genet. Metab..

[31] Zikanova M., Skopova V., Hnizda A., Krijt J., Kmoch S. (2010). Biochemical and structural analysis of 14 mutant adsl enzyme complexes and correlation to phenotypic heterogeneity of adenylosuccinate lyase deficiency. Hum. Mutat..

[32] Sabina R.L., Holmes E.W., Becker M.A. (1984). The Enzymatic Synthesis of 5-Amino-4-Imidazolecarboxamide Riboside Triphosphate (ZTP). Science.

[33] Daignan-Fornier B., Pinson B. (2012). 5-Aminoimidazole-4-carboxamide-1-beta-D-ribofuranosyl 5’-Monophosphate (AICAR), a Highly Conserved Purine Intermediate with Multiple Effects. Metabolites.

[34] Stone T.W., Simmonds H.A. (1991). Purines: Basic and Clinical Aspects.

[35] van den Bergh F., Vincent M.F., Jaeken J., van den Bergh G. (1993). Residual adenylosuccinase activities in fibroblasts of adenylosuccinase-deficient children: Parallel deficiency with adenylosuccinate and succinyl-AICAR in profoundly retarded patients and non-parallel deficiency in a mildly retarded girl. J. Inherit. Metab. Dis..

[36] Atkinson D.E., Walton G.M. (1967). Adenosine Triphosphate Conservation in Metabolic Regulation. J. Biol. Chem..

[37] Shen L., Fall L., Walton G.M., Atkinson D.E. (1968). Interaction between energy charge and metabolite modulation in the regulation of enzymes of amphibolic sequences. Phosphofructokinase and pyruvate dehydrogenase. Biochemistry.

[38] Swedes J.S., Sedo R.J., E Atkinson D. (1975). Relation of growth and protein synthesis to the adenylate energy charge in an adenine-requiring mutant of Escherichia coli. J. Biol. Chem..

[39] De La Fuente I.M., Cortes J.M., Valero E., Desroches M., Rodrigues S., Malaina I., Martínez L. (2014). On the Dynamics of the Adenylate Energy System: Homeorhesis vs. Homeostasis. PLoS ONE.

[40] Boutchueng-Djidjou M., Collard-Simard G., Fortier S., Hébert S., Kelly I., Landry C.R., Faure R.L. (2015). The Last Enzyme of the De Novo Purine Synthesis Pathway 5-aminoimidazole-4-carboxamide Ribonucleotide Formyltransferase/IMP Cyclohydrolase (ATIC) Plays a Central Role in Insulin Signaling and the Golgi/Endosomes Protein Network. Mol. Cell. Proteom..

[41] Kit S. (1955). The biosynthesis of free glycine and serine by tumors. Cancer Res..

[42] Tedeschi P.M., Markert E.K., Gounder M., Lin H., Dvorzhinski D., Dolfi S.C., Chan L.L.-Y., Qiu J., DiPaola R.S., Hirshfield K.M. (2013). Contribution of serine, folate and glycine metabolism to the ATP, NADPH and purine requirements of cancer cells. Cell Death Dis..

[43] Locasale J.W., Grassian A.R., Melman T., Lyssiotis C.A., Mattaini K.R., Bass A.J., Heffron G., Metallo C.M., Muranen T., Sharfi H. (2011). Phosphoglycerate dehydrogenase diverts glycolytic flux and contributes to oncogenesis. Nat. Genet..

[44] Zogg C.K. (2014). Phosphoglycerate Dehydrogenase: Potential Therapeutic Target and Putative Metabolic Oncogene. J. Oncol..

[45] Martínez-Martín D., Martinez-Martin N., Blas-García A., Morales J.M., Martí-Cabrera M., Monleon D., Apostolova N. (2012). Metabolomics of the effect of AMPK activation by AICAR on human umbilical vein endothelial cells. Int. J. Mol. Med..

[46] Li S., Gao D., Jiang Y. (2019). Function, Detection and Alteration of Acylcarnitine Metabolism in Hepatocellular Carcinoma. Metabolites.

[47] Tai E.S., Tan M.L.S., Stevens R.D., Low Y.L., Muehlbauer M.J., Goh D.L.M., Ilkayeva O.R., Wenner B.R., Bain J.R., Lee J.J.M. (2010). Insulin resistance is associated with a metabolic profile of altered protein metabolism in Chinese and Asian-Indian men. Diabetologia.

[48] Corton J.M., Gillespie J.G., Hawley S.A., Hardie D.G. (1995). 5-Aminoimidazole-4-Carboxamide Ribonucleoside. A Specific Method for Activating AMP-Activated Protein Kinase in Intact Cells?. Eur. J. Biochem..

[49] Koves T., Ussher J.R., Noland R.C., Slentz D., Mosedale M., Ilkayeva O., Bain J.R., Stevens R., Dyck J.R., Newgard C.B. (2008). Mitochondrial Overload and Incomplete Fatty Acid Oxidation Contribute to Skeletal Muscle Insulin Resistance. Cell Metab..

[50] Donti T.R., Cappuccio G., Hubert L., Neira J., Atwal P.S., Miller M.J., Cardon A.L., Sutton V.R., Porter B.E., Baumer F. (2016). Diagnosis of adenylosuccinate lyase deficiency by metabolomic profiling in plasma reveals a phenotypic spectrum. Mol. Genet. Metab. Rep..

[51] Subedi A., Muroi M., Futamura Y., Kawamura T., Aono H., Nishi M., Ryo A., Watanabe N., Osada H. (2019). A novel inhibitor of tumorspheres reveals the activation of the serine biosynthetic pathway upon mitochondrial inhibition. FEBS Lett..

[52] Labuschagne C.F., Van Den Broek N.J.F., Mackay G.M., Vousden K.H., Maddocks O.D.K. (2014). Serine, but Not Glycine, Supports One-Carbon Metabolism and Proliferation of Cancer Cells. Cell Rep..

[53] Ulrey C.L., Liu L., Andrews L.G., Tollefsbol T.O. (2005). The impact of metabolism on DNA methylation. Hum. Mol. Genet..

[54] Lu S.C. (2000). S-Adenosylmethionine. Int. J. Biochem. Cell Biol..

[55] Kim P.B., Nelson J.W., Breaker R.R. (2015). An Ancient Riboswitch Class in Bacteria Regulates Purine Biosynthesis and One-Carbon Metabolism. Mol. Cell.

[56] Ducker G., Rabinowitz J.D. (2015). ZMP: A Master Regulator of One-Carbon Metabolism. Mol. Cell.

[57] Wojtowicz P., Zrostlíková J., Veronika Š., Dostálová E., Žídková L., Bruheim P., Adam T. (2008). Comprehensive Two-Dimensional Gas Chromatography Coupled to Time-of-Flight Mass Spectrometry in Human Metabolomics. Gas Chromatography.

[58] Sumner L.W., Amberg A., Barrett D., Beale M.H., Beger R., Daykin C.A., Fan T.W.-M., Fiehn O., Goodacre R., Griffin J.L. (2007). Proposed minimum reporting standards for chemical analysis. Chemical Analysis Working Group (CAWG) Metabolomics Standards Initiative (MSI). Metabolomics.

[59] Karlíková R., Široká J., Friedecký D., Faber E., Hrdá M., Mičová K., Fikarová I., Gardlo A., Janečková H., Vrobel I. (2016). Metabolite Profiling of the Plasma and Leukocytes of Chronic Myeloid Leukemia Patients. J. Proteome Res..

[60] Cleveland W.S. (1979). Robust Locally Weighted Regression and Smoothing Scatterplots. J. Am. Stat. Assoc..

[61] Dunn W.B., Broadhurst D., Begley P., Zelena E., Francis-McIntyre S., Anderson N., Brown M., Knowles J.D., Halsall A., Haselden J.N. (2011). Procedures for large-scale metabolic profiling of serum and plasma using gas chromatography and liquid chromatography coupled to mass spectrometry. Nat. Protoc..

[62] Pawlowsky-Glahn V., Egozcue J.J., Tolosana-Delgado R. (2015). Modelling and Analysis of Compositional Data.

