# Supplementary material for: Combined Targeted and Untargeted Profiling of HeLa Cells Deficient in Purine De Novo Synthesis
Source: Metabolites. 2022 Mar 13;12(3):241. doi: 10.3390/metabo12030241 (PMC8948957; doi:10.3390/metabo12030241)
Supplement: Supplementary file 1 [file metabolites-12-00241-s001.zip › metabolites-1588679-supplementary.pdf]

Figure S1: Compound Discoverer workflow parameters.

Created with Discoverer version: 3.0.0.294

[Input Files (0)]

-->Select Spectra (1)

[Select Spectra (1)]

-->Align Retention Times (2)

[Align Retention Times (2)]

-->Detect Compounds (3)

[Detect Compounds (3)]

-->Group Compounds (4)

[Group Compounds (4)]

-->Fill Gaps (16)

-->Search ChemSpider (8)

-->Assign Compound Annotations (9)

-->Predict Compositions (12)

-->Map to Metabolika Pathways (22)

-->Search mzCloud (21)

[Fill Gaps (16)]

-->Normalize Areas (19)

[Normalize Areas (19)]

-->Mark Background Compounds (15)

[Mark Background Compounds (15)]

[Search ChemSpider (8)]

[Assign Compound Annotations (9)]

[Predict Compositions (12)]

[Map to Metabolika Pathways (22)]

[Search mzCloud (21)]

[Differential Analysis (10)]

[Descriptive Statistics (18)]

Processing node 0: Input Files

Input Data:

- File Name(s) (Hidden):

C:\CRISPR_DATA\1_QC1_2.raw

C:\CRISPR_DATA\2_PFAS_G6_4n_2.raw

C:\CRISPR_DATA\3_ADSL_D4_4n_2.raw

C:\CRISPR_DATA\4_PAICS_3_2.raw

C:\CRISPR_DATA\5_CON1_6n_2.raw

C:\CRISPR_DATA\6_QC2_2.raw

C:\CRISPR_DATA\11_QC3_2.raw

C:\CRISPR_DATA\13_ATIC_G4_2_2.raw

C:\CRISPR_DATA\16_QC4_2.raw

C:\CRISPR_DATA\19_GART_2_2.raw

C:\CRISPR_DATA\21_QC5_2.raw

C:\CRISPR_DATA\23_PAICS_5n_2.raw

C:\CRISPR_DATA\24_GART_5n_2.raw

C:\CRISPR_DATA\25_PFAS_G6_2_2.raw

C:\CRISPR_DATA\26_QC6_2.raw

C:\CRISPR_DATA\27_CON1_3n_2.raw

C:\CRISPR_DATA\28_ADSL_D4_2_2.raw

C:\CRISPR_DATA\31_QC7_2.raw

C:\CRISPR_DATA\33_CON1_1n_2.raw

C:\CRISPR_DATA\35_ATIC_G4_3_2.raw

C:\CRISPR_DATA\36_QC8_2.raw

C:\CRISPR_DATA\37_PFAS_G6_3_180614134308_2.raw

C:\CRISPR_DATA\38_ATIC_G4_4_2.raw

C:\CRISPR_DATA\41_QC9_2.raw

C:\CRISPR_DATA\43_ADSL_D4_5_2.raw

C:\CRISPR_DATA\46_QC10_2.raw

C:\CRISPR_DATA\51_QC11_2.raw

C:\CRISPR_DATA\55_GART_1_2.raw

C:\CRISPR_DATA\56_QC12_2.raw

C:\CRISPR_DATA\58_CON1_2n_2.raw

C:\CRISPR_DATA\60_PAICS_1_180614232857_2.raw

C:\CRISPR_DATA\61_QC13_2.raw

C:\CRISPR_DATA\62_ADSL_D4_6_2.raw

C:\CRISPR_DATA\63_GART_3_180615210440_2.raw

C:\CRISPR_DATA\65_ADSL_D4_1_2.raw

C:\CRISPR_DATA\66_QC14_2.raw

C:\CRISPR_DATA\70_PAICS_2_2.raw

C:\CRISPR_DATA\71_QC15_2.raw

C:\CRISPR_DATA\75_CON1_4n_2.raw

C:\CRISPR_DATA\76_QC16_2.raw

C:\CRISPR_DATA\79_ATIC_G4_6_2.raw

C:\CRISPR_DATA\80_PFAS_G6_6_2.raw

C:\CRISPR_DATA\81_QC17_2.raw

C:\CRISPR_DATA\83_PFAS_G6_1_2.raw

C:\CRISPR_DATA\84_ATIC_G4_5_2.raw

C:\CRISPR_DATA\85_GART_6_2.raw

C:\CRISPR_DATA\86_QC18_2.raw

C:\CRISPR_DATA\90_PAICS_4_2.raw

C:\CRISPR_DATA\91_QC19_2.raw

C:\CRISPR_DATA\92_PAICS_6_2.raw

C:\CRISPR_DATA\94_ADSL_D4_3_180615152149_2.raw

C:\CRISPR_DATA\95_GART_4_2.raw

C:\CRISPR_DATA\96_QC20_2.raw

C:\CRISPR_DATA\97_ATIC_G4_1_2.raw

C:\CRISPR_DATA\98_CON1_5n_2.raw

C:\CRISPR_DATA\101_QC21_2.raw

C:\CRISPR_DATA\102_PFAS_G6_5_2.raw

C:\CRISPR_DATA\106_QC22_2.raw

C:\CRISPR_DATA\107_QC23_2.raw

C:\CRISPR_DATA\108_QC24_2.raw

C:\CRISPR_DATA\109_QC25_2.raw

C:\CRISPR_DATA\110_QC26_2.raw

C:\CRISPR_DATA\BLANK17_1_2.raw

C:\CRISPR_DATA\BLANK17_2_2.raw

C:\CRISPR_DATA\BLANK17_3_2.raw

C:\CRISPR_DATA\BLANK18_1_2.raw

C:\CRISPR_DATA\BLANK18_2_2.raw

C:\CRISPR_DATA\DDA_150_250.raw

C:\CRISPR_DATA\DDA_250_350.raw

C:\CRISPR_DATA\DDA_350_450.raw

C:\CRISPR_DATA\DDA_450_1500.raw

Processing node 1: Select Spectra

----

1. General Settings:

- Precursor Selection: Use MS(n - 1) Precursor

- Use Isotope Pattern in Precursor Reevaluation: True

- Provide Profile Spectra: Automatic

- Store Chromatograms: False

2. Spectrum Properties Filter:

- Lower RT Limit: 1

- Upper RT Limit: 17
- First Scan: 0
- Last Scan: 0
- Ignore Specified Scans: (not specified)
- Lowest Charge State: 0
- Highest Charge State: 0
- Min. Precursor Mass: 70 Da
- Max. Precursor Mass: 5000 Da
- Total Intensity Threshold: 0
- Minimum Peak Count: 1

3. Scan Event Filters:

- Mass Analyzer: (not specified)
- MS Order: Any
- Activation Type: (not specified)
- Min. Collision Energy: 0
- Max. Collision Energy: 1000
- Scan Type: Any
- Polarity Mode: (not specified)

4. Peak Filters:

- S/N Threshold (FT-only): 0

5. Replacements for Unrecognized Properties:

- Unrecognized Charge Replacements: 1
- Unrecognized Mass Analyzer Replacements: ITMS
- Unrecognized MS Order Replacements: MS2
- Unrecognized Activation Type Replacements: CID
- Unrecognized Polarity Replacements: +

- Unrecognized MS Resolution@200 Replacements: 60000

- Unrecognized MSn Resolution@200 Replacements: 30000

Processing node 2: Align Retention Times

----

1. General Settings:

- Alignment Model: Adaptive curve

- Alignment Fallback: Use Linear Model

- Maximum Shift [min]: 0.8

- Shift Reference File: True

- Mass Tolerance: 5 ppm

- Remove Outlier: True

Processing node 3: Detect Compounds

----

1. General Settings:

- Mass Tolerance [ppm]: 5 ppm

- Intensity Tolerance [%]: 30

- S/N Threshold: 1.5

- Min. Peak Intensity: 10000

- Ions:

    [2M+ACN+H]+1

    [2M+ACN+Na]+1

    [2M+H]+1

    [2M+K]+1

    [2M+Na]+1

    [2M+NH4]+1

    [M+ACN+H]+1

    [M+ACN+Na]+1

[M+H]+1

[M+K]+1

[M+Na]+1

[M+NH4]+1

- Base Ions: [M+H]+1
- Min. Element Counts: C H
- Max. Element Counts: C150 H250 Br3 Cl4 K2 N10 Na2 O18 P5 S5

2. Peak Detection:

- Filter Peaks: True
- Max. Peak Width [min]: 0.5
- Remove Singlets: True
- Min. # Scans per Peak: 8
- Min. # Isotopes: 2

Processing node 4: Group Compounds

----

1. Compound Consolidation:

- Mass Tolerance: 5 ppm
- RT Tolerance [min]: 0.5

2. Fragment Data Selection:

- Preferred Ions: [M+H]+1

Processing node 16: Fill Gaps

----

1. General Settings:

- Mass Tolerance: 5 ppm
- S/N Threshold: 1.5

- Use Real Peak Detection: True

Processing node 19: Normalize Areas

----

1. QC-based Area Correction:

- Regression Model: Linear

- Min. QC Coverage [%]: 25

- Max. QC Area RSD [%]: 50

- Max. # Files Between QC Files: 20

2. Area Normalization:

- Normalization Type: None

- Exclude Blanks: True

Processing node 15: Mark Background Compounds

----

1. General Settings:

- Max. Sample/Blank: 5

- Max. Blank/Sample: 0

- Hide Background: True

Processing node 8: Search ChemSpider

----

1. Search Settings:

- Database(s): Human Metabolome Database; KEGG; LipidMAPS; PubMed

- Search Mode: By Formula or Mass

- Mass Tolerance: 5 ppm

- Max. # of results per compound: 100

- Max. # of Predicted Compositions to be searched per Compound: 3

- Result Order (for Max. # of results per compound): Order By Reference Count (DESC)

2. Predicted Composition Annotation:

- Check All Predicted Compositions: False

Processing node 9: Assign Compound Annotations

----

1. General Settings:

- Mass Tolerance: 5 ppm

2. Data Sources:

- Data Source #1: Predicted Compositions

- Data Source #2: ChemSpider Search

- Data Source #3: (not specified)

- Data Source #4: (not specified)

- Data Source #5: (not specified)

Processing node 12: Predict Compositions

----

1. Prediction Settings:

- Mass Tolerance: 5 ppm

- Min. Element Counts: C H

- Max. Element Counts: C250 H190 N10 O18 P5 S5

- Min. RDBE: 0

- Max. RDBE: 40

- Min. H/C: 0.1

- Max. H/C: 3.5

- Max. # Candidates: 10

- Max. # Internal Candidates: 200

2. Pattern Matching:

- Intensity Tolerance [%]: 30

- Intensity Threshold [%]: 0.1

- S/N Threshold: 3

- Min. Spectral Fit [%]: 30

- Min. Pattern Cov. [%]: 90

- Use Dynamic Recalibration: True

3. Fragments Matching:

- Use Fragments Matching: True

- Mass Tolerance: 10 ppm

- S/N Threshold: 3

Processing node 22: Map to Metabolika Pathways

----

1. Search Settings:

- Metabolika Pathways:

- Search Mode: By Formula or Mass

2. By Mass Search Settings:

- Mass Tolerance: 5 ppm

3. By Formula Search Settings:

- Max. # of Predicted Compositions to be searched per Compound: 3

4. Display Settings:

- Max. # Pathways in 'Pathways' column: 20

Processing node 21: Search mzCloud

----

1. Search Settings:

- Compound Classes: All

- Match Ion Activation Type: True

- Match Ion Activation Energy: Match with Tolerance

- Ion Activation Energy Tolerance: 20

- Apply Intensity Threshold: True

- Precursor Mass Tolerance: 10 ppm

- FT Fragment Mass Tolerance: 10 ppm

- IT Fragment Mass Tolerance: 0.4 Da

- Identity Search: HighChem HighRes

- Similarity Search: Similarity Reverse

- Library: Reference

- Post Processing: Recalibrated

- Match Factor Threshold: 60

- Max. # Results: 10

Processing node 10: Differential Analysis

----

1. General Settings:

- Log10 Transform Values: True

Processing node 18: Descriptive Statistics

No parameters

This file contains the following filters:

Row Filter for Compounds:

AND

|

+--BackgroundStatus is false

|

+--NormArea in any category

|

+--Area (Max.) is greater than 10000.00

|

+--# Usable QC is greater than 18

|

+--Group CV [%] in every category

|

+--RSD QC Areas [%] is less than 30.00

|

+--Formula is not blank

Table S1: Filters used in CD for feature number reduction.

Background	Is false
Area (Max.)	Is greater than 4000
Group CV (%)	Is less than 150 in every sample group
RSD QC Areas (%)	Is less than 30 %
Formula	Is not blank

Table S2: The top 20 most significant features of VIP-OPLS-DA detected in crGART cells compared to controls in untargeted analysis. Abbreviations: PC – phosphatidylcholine. PS – phosphatidylserine. 7-MG – 7-methylguanine. GLC-6P – glucose-6-phosphate

order of significance	RT (min)	detected m/z	theoretical m/z	identification	mean VIP	SD VIP	fold change	p-value
1	3.58	555.3909		PC	3.72	0.09	-3.59	5.90E-07
2	3.66	368.3295	-	unknown	3.18	0.06	-2.92	2.35E-07
3	5.23	375.1219	-	unknown	2.87	0.13	-2.26	3.52E-03
4	7.25	141.0191	-	unknown	2.85	0.13	-2.02	1.06E-04
5	4.39	823.5377	823.5363	PS (39:0)	2.84	0.08	-2.18	7.07E-07
6	7.78	338.0632	338.0628	AICAR	2.71	0.10	-1.92	2.53E-03
7	3.64	904.4673	-	unknown	2.57	0.07	1.75	1.33E-06
8	5.00	149.0064	-	unknown	2.55	0.22	2.55	3.44E-03
9	3.44	551.3955	551.3951	PC (20:0)	2.52	0.12	-1.82	1.42E-05
10	4.14	278.0409	-	unknown	2.47	0.14	-1.56	1.83E-04
11	3.50	495.3359	-	unknown	2.43	0.08	-1.42	7.22E-07
12	7.68	126.0317	-	unknown	2.40	0.08	-1.45	1.61E-08
13	4.21	165.0651	165.0651	7-mG	2.40	0.12	-1.89	2.73E-04
14	8.49	291.9755	-	unknown	2.38	0.10	1.57	3.47E-07
15	6.00	287.0681	-	unknown	2.37	0.09	1.90	1.38E-04
16	4.82	413.0010	-	unknown	2.27	0.09	1.39	1.23E-05
17	3.79	863.5916	-	unknown	2.26	0.17	2.22	1.30E-02
18	7.77	260.0303	260.0297	GLC-6P	2.22	0.07	-1.23	3.03E-07
19	3.60	675.5240	-	unknown	2.21	0.08	-1.28	9.78E-05
20	3.48	142.1106	-	unknown	2.19	0.11	1.41	6.62E-04

Table S3: The top 20 most significant features of VIP-OPLS-DA detected in crPFAS cells compared to controls in untargeted analysis. Abbreviations: PS – phosphatidylserine. ala-gln - alanyl-glutamine

order of significance	RT (min)	detected m/z	theoretical m/z	identification	mean VIP	SD VIP	fold change	p-value
1	7.76	314.0520	314.0515	FGAR	4.02	0.07	6.09	4.00E-15
2	9.69	473.9849	473.9842	FGAr-3P	3.96	0.07	5.89	1.82E-15
3	3.81	234.0855	234.0852	FGAr	3.76	0.07	5.30	4.57E-12
4	8.56	394.0186	394.0178	FGAr-2P	3.25	0.06	4.00	4.06E-13
5	3.66	368.3295	-	unknown	2.73	0.04	-3.12	1.51E-07
6	4.45	161.0688	-	unknown	2.63	0.07	-2.79	1.06E-06
7	5.23	375.1219	-	unknown	2.52	0.12	-2.69	1.91E-03
8	4.30	755.4238	-	unknown	2.50	0.05	2.31	1.08E-05
9	7.25	141.0191	-	unknown	2.46	0.08	-2.17	1.01E-04
10	4.39	823.5377	823.5363	PS (39:0)	2.43	0.04	-2.42	2.35E-06
11	4.41	757.4909	-	unknown	2.41	0.04	-2.37	2.67E-07
12	3.60	675.5240	-	unknown	2.39	0.04	-2.17	7.26E-11
13	4.27	783.4562	-	unknown	2.36	0.06	2.08	1.20E-07
14	3.50	495.3359	-	unknown	2.23	0.06	-1.74	4.68E-07
15	5.56	217.1069	217.1063	ala-gln	2.22	0.12	-1.92	1.82E-04
16	7.78	338.0632	338.0628	AICAR	2.21	0.10	-1.96	2.46E-03
17	6.52	488.1085	-	unknown	2.16	0.06	-1.85	9.32E-05
18	7.68	96.0211	96.0211	fragment of FGAR	2.13	0.05	1.69	1.56E-08
19	4.69	159.1259	-	unknown	2.10	0.05	1.63	6.13E-08
20	7.68	126.0317	-	unknown	2.03	0.04	-1.55	1.36E-08

Table S4: The top 20 most significant features of VIP-OPLS-DA detected in crPAICS cells compared to controls in untargeted analysis. Abbreviations: PC – phosphatidylcholine. PS – phosphatidylserine. 5-MC – 5-methylcytidine.

order of significance	RT (min)	detected m/z	theoretical m/z	identification	mean VIP	SD VIP	fold change	p-value
1	3.74	215.0908	215.0906	AIr	4.32	0.07	6.96	7.23E-14
2	7.80	295.0575	295.0569	AIR	3.54	0.06	4.64	6.53E-08
3	4.05	173.0701	-	unknown	3.44	0.06	4.41	3.74E-07
4	4.15	233.1016	233.1012	FGAMr	3.33	0.06	4.01	9.72E-06
5	8.49	715.1900	-	unknown	3.13	0.05	3.62	2.58E-12
6	3.58	257.1015	257.1012	5-mC	3.11	0.06	3.69	3.39E-08
7	3.58	555.3909	-	PC	3.09	0.06	-3.72	2.77E-08
8	4.31	454.1822	-	unknown	3.08	0.07	3.58	4.13E-08
9	3.81	234.0855	234.0852	FGAr	2.91	0.05	3.18	6.52E-11
10	7.25	141.0191	-	unknown	2.83	0.09	-2.97	7.34E-06
11	3.66	368.3295	-	unknown	2.75	0.03	-3.09	1.33E-07
12	7.33	313.0682	313.0675	FGAMR	2.70	0.04	2.73	2.60E-10
13	4.71	328.1392	-	unknown	2.70	0.12	3.12	3.66E-04
14	3.84	539.4194	-	unknown	2.60	0.05	2.53	4.32E-10
15	4.10	377.3046	-	unknown	2.47	0.06	-2.36	6.74E-06
16	4.21	165.0651	165.0651	7-mG	2.41	0.06	-2.41	2.04E-06
17	4.86	528.3024	-	unknown	2.34	0.06	2.00	6.95E-07
18	3.44	551.3955	551.3951	PC (20:0)	2.27	0.08	-2.07	6.04E-06
19	4.39	823.5377	823.5363	PS (39:0)	2.24	0.05	-2.04	1.44E-06
20	5.23	375.1219	-	unknown	2.19	0.18	-2.39	5.10E-03

Table S5: The top 20 most significant features of VIP-OPLS-DA detected in crADSL cells compared to controls in untargeted analysis. Abbreviations: PC – phosphatidylcholine. 7-MG – 7-methylguanine.

order of significance	RT (min)	detected m/z	theoretical m/z	identification	mean VIP	SD VIP	fold change	p-value
1	9.44	454.0744	454.0737	SAICAR	3.84	0.12	5.81	7.06E-07
2	7.76	374.1079	374.1074	SAICAr	3.79	0.12	5.74	3.72E-07
3	3.58	555.3909	-	PC	3.03	0.08	-3.52	1.21E-08
4	7.76	383.1079	383.1077	SAdo	2.86	0.07	3.28	1.72E-11
5	9.43	463.0745	463.0740	S-AMP	2.75	0.07	2.90	8.90E-10
6	3.74	215.0908	215.0906	AIr	2.70	0.12	3.08	5.20E-05
7	3.66	368.3295	-	unknown	2.56	0.04	-2.74	9.93E-07
8	5.23	375.1219	-	unknown	2.47	0.10	-2.54	2.37E-03
9	3.76	564.5127	-	unknown	2.42	0.06	-2.32	1.47E-06
10	6.00	287.0681	-	unknown	2.33	0.04	2.49	2.01E-05
11	4.21	165.0651	165.0651	7-mG	2.33	0.06	-1.52	4.72E-06
12	3.44	551.3955	551.3951	PC (20:0)	2.29	0.08	-2.12	7.69E-06
13	6.03	379.1599	-	unknown	2.23	0.09	2.56	3.37E-04
14	4.39	823.5377	823.5363	PS (39:0)	2.22	0.05	-2.23	1.57E-06
15	4.06	538.2708	-	unknown	2.17	0.05	1.83	8.58E-05
16	3.64	904.4673	-	unknown	2.16	0.06	1.82	6.79E-07
17	7.78	338.0632	338.0628	AICAR	2.16	0.08	-1.82	3.16E-03
18	5.00	149.0064	-	unknown	2.16	0.13	2.56	3.20E-03
19	3.47	509.3853	509.3845	PC (18:0)	2.14	0.07	-1.89	9.40E-07
20	4.10	377.3046	-	unknown	2.13	0.07	-1.82	1.59E-06

Table S6: The top 20 most significant features of VIP-OPLS-DA detected in crATIC cells compared to controls in untargeted analysis. Abbreviations: PC – phosphatidylcholine. PS – phosphatidylserine. 7-MG – 7-methylguanine. ala-gln – alanyl-glutamine. NAD+ - nicotinamide adenine dinucleotide

order of significance	RT (min)	detected m/z	theoretical m/z	identification	mean VIP	SD VIP	fold change	p-value
1	3.58	555.3909	-	PC	3.01	0.08	-3.75	2.80E-07
2	6.09	222.0680	222.0674	L-cystathionine	2.96	0.07	-3.75	1.22E-07
3	6.03	379.1599	-	unknown	2.78	0.03	3.57	2.52E-05
4	3.91	258.0966	258.0964	AICAr	2.68	0.12	3.25	1.07E-04
5	3.66	368.3295	-	unknown	2.59	0.05	-3.00	3.10E-07
6	9.44	454.0744	454.0737	SAICAR	2.54	0.09	2.74	1.91E-05
7	7.78	338.0632	338.0628	AICAR	2.52	0.13	2.98	6.19E-04
8	4.18	396.1587	-	unknown	2.41	0.08	2.53	2.06E-07
9	7.76	374.1079	374.1074	SAICAr	2.41	0.09	2.51	1.95E-05
10	5.56	217.1069	217.1063	ala-gln	2.39	0.06	2.78	1.48E-05
11	4.21	165.0651	165.0651	7-mG	2.36	0.07	-2.43	1.28E-05
12	3.48	142.1106	-	unknown	2.33	0.11	-2.68	8.13E-05
13	5.00	149.0064	-	unknown	2.31	0.09	2.77	4.04E-04
14	4.05	179.0793	-	unknown	2.27	0.08	2.56	4.04E-04
15	3.84	539.4194	-	unknown	2.27	0.07	2.10	3.06E-05
16	4.39	823.5377	823.5363	PS (39:0)	2.17	0.03	-2.03	2.70E-06
17	4.10	377.3046	-	unknown	2.17	0.07	-1.95	2.56E-06
18	7.78	664.0942	664.0931	deamino-NAD^+	2.16	0.06	-1.88	6.77E-07
19	6.15	308.1226	-	unknown	2.13	0.08	2.32	6.68E-04
20	10.81	497.9963	497.9954	AICAr-3P	2.10	0.16	2.03	3.21E-03

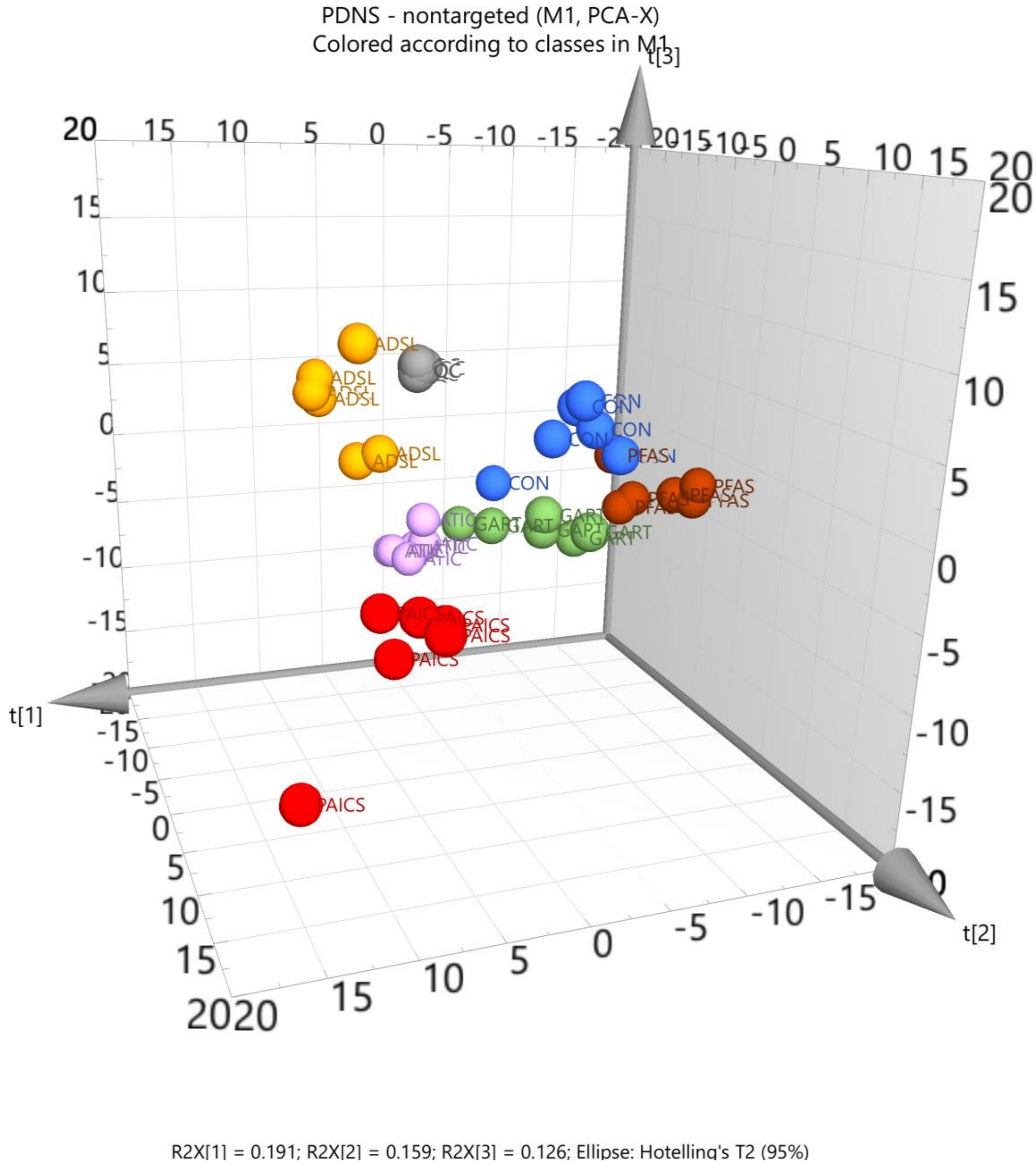

Figure S2: 3D Principal component analysis of HeLa deficient and control cells that were subjected to an untargeted metabolomic analysis. Slight separation of crGART and control cells is probably not connected to the metabolites involved in PDNS (See Table S2 in Supplemetary data). Grey dots represent QC samples.

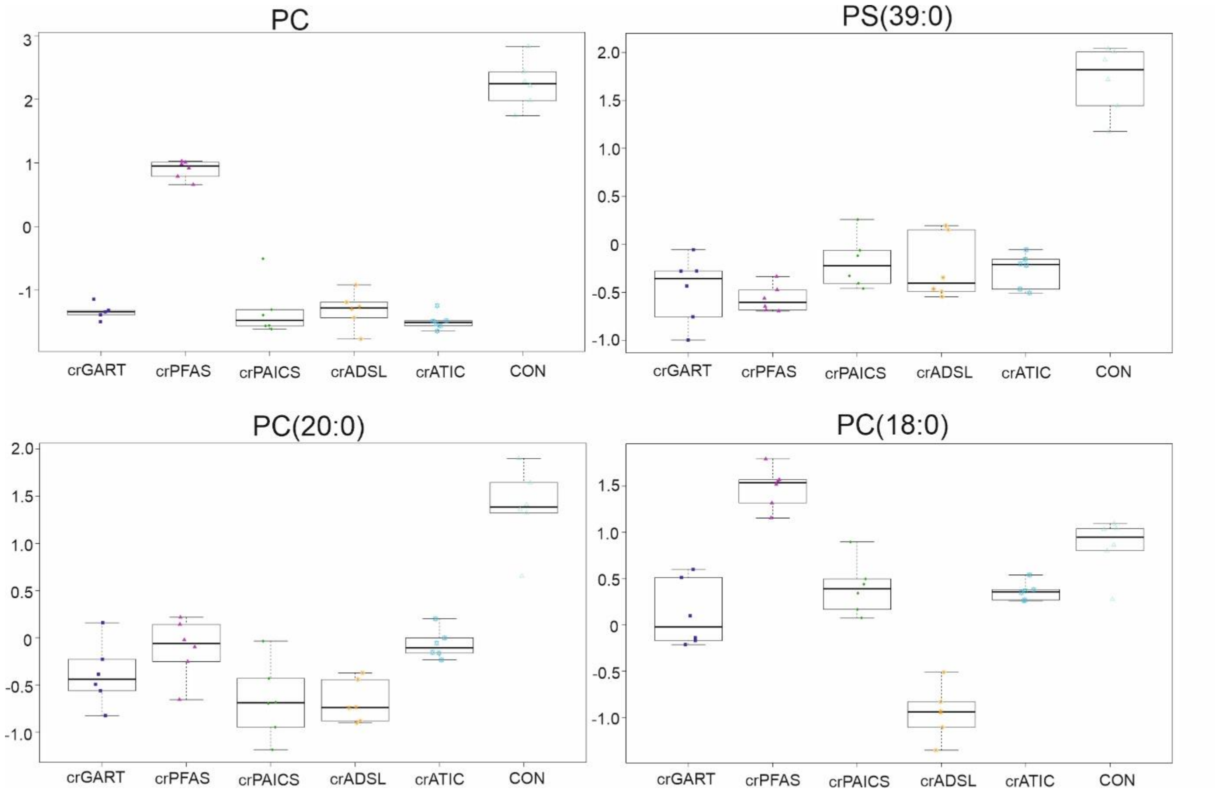

Figure S3: Boxplots of significant lipids identified in all cell lines.

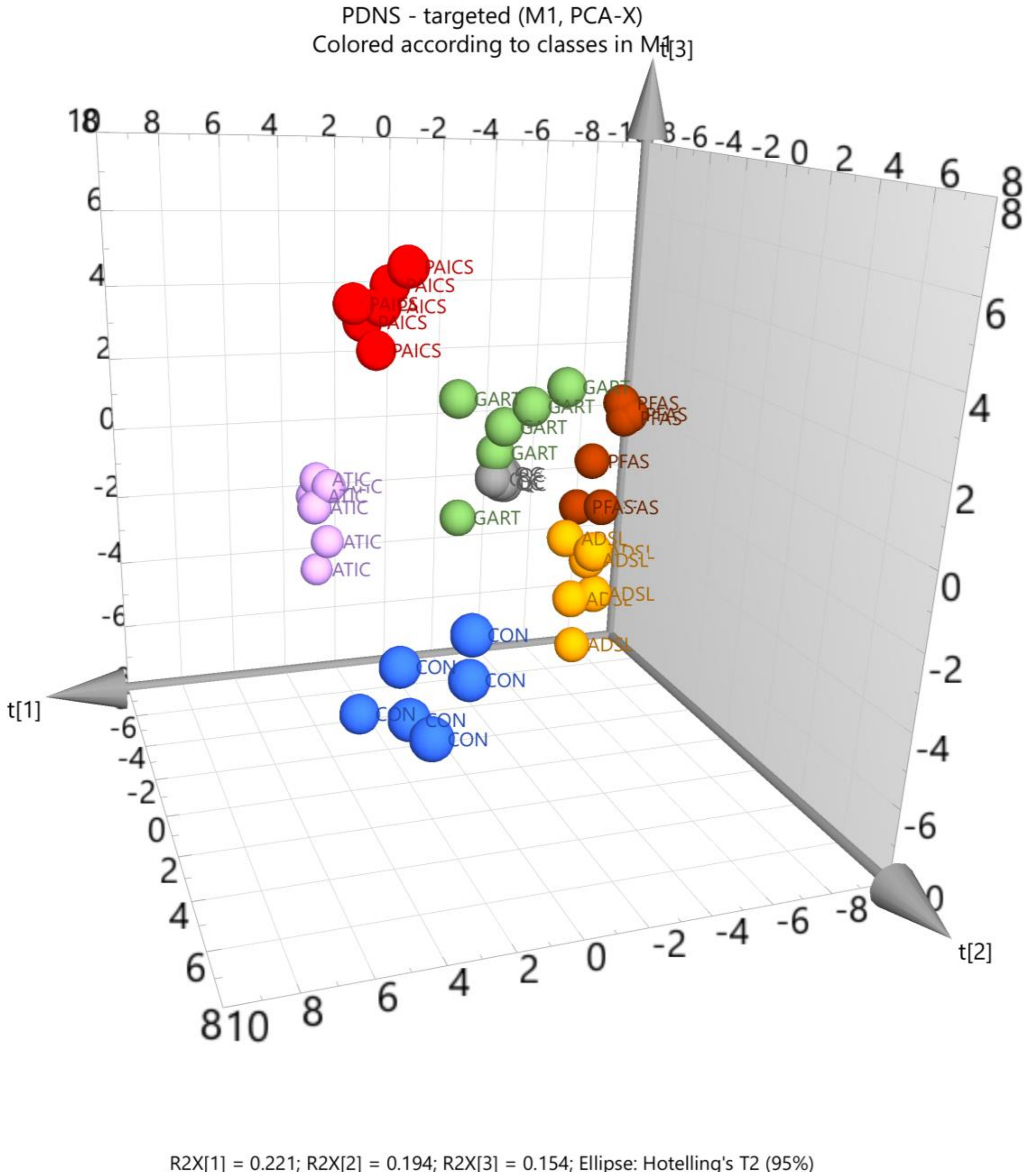

Figure S4: 3D Principal component analysis of HeLa deficient and control cells that were subjected to a targeted metabolomic analysis. Grey dots represent QC samples.

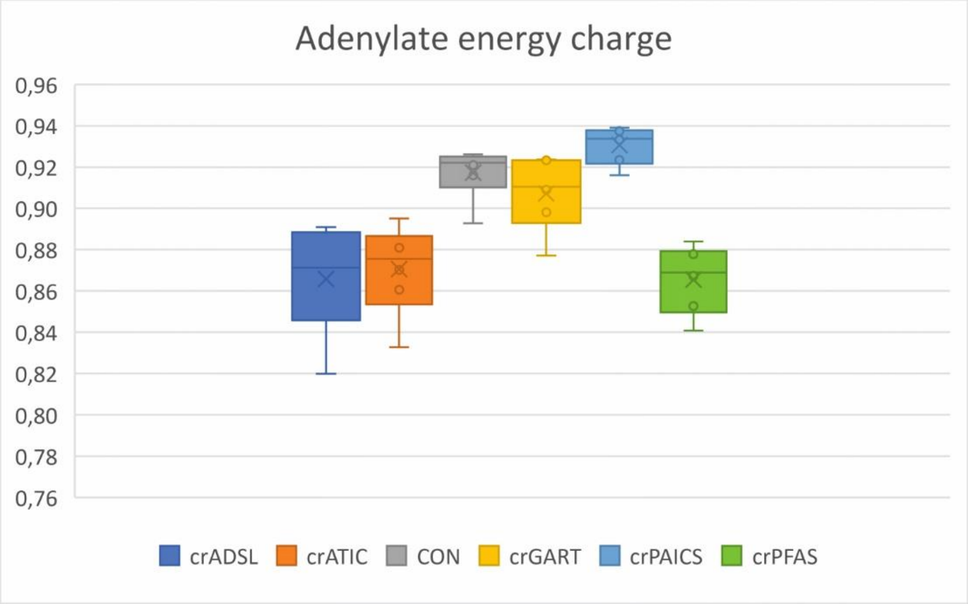

Figure S5: The adenylate energy charge plot shows a comparison of box plots of deficient cell lines and controls. We calculated the Wilcoxon rank-sum test for a two-tailed hypothesis to cover the experimental variability and a left-tailed test (i.e., the alternative hypothesis states the lower energy charge of the deficient cell lines) to estimate the biological influence of the defects. A nonparametric approach to hypothesis testing was chosen since the normality of the controls was rejected. Although the AEC of crADSL, crATIC, and crPFAS cells was significantly lowered ($p < 0.01$; Bonferroni correction was applied to the level of significance based on multiple testing), the crPAICS cell line exhibited higher AEC pointing to experimental variability in line-to line behavior.

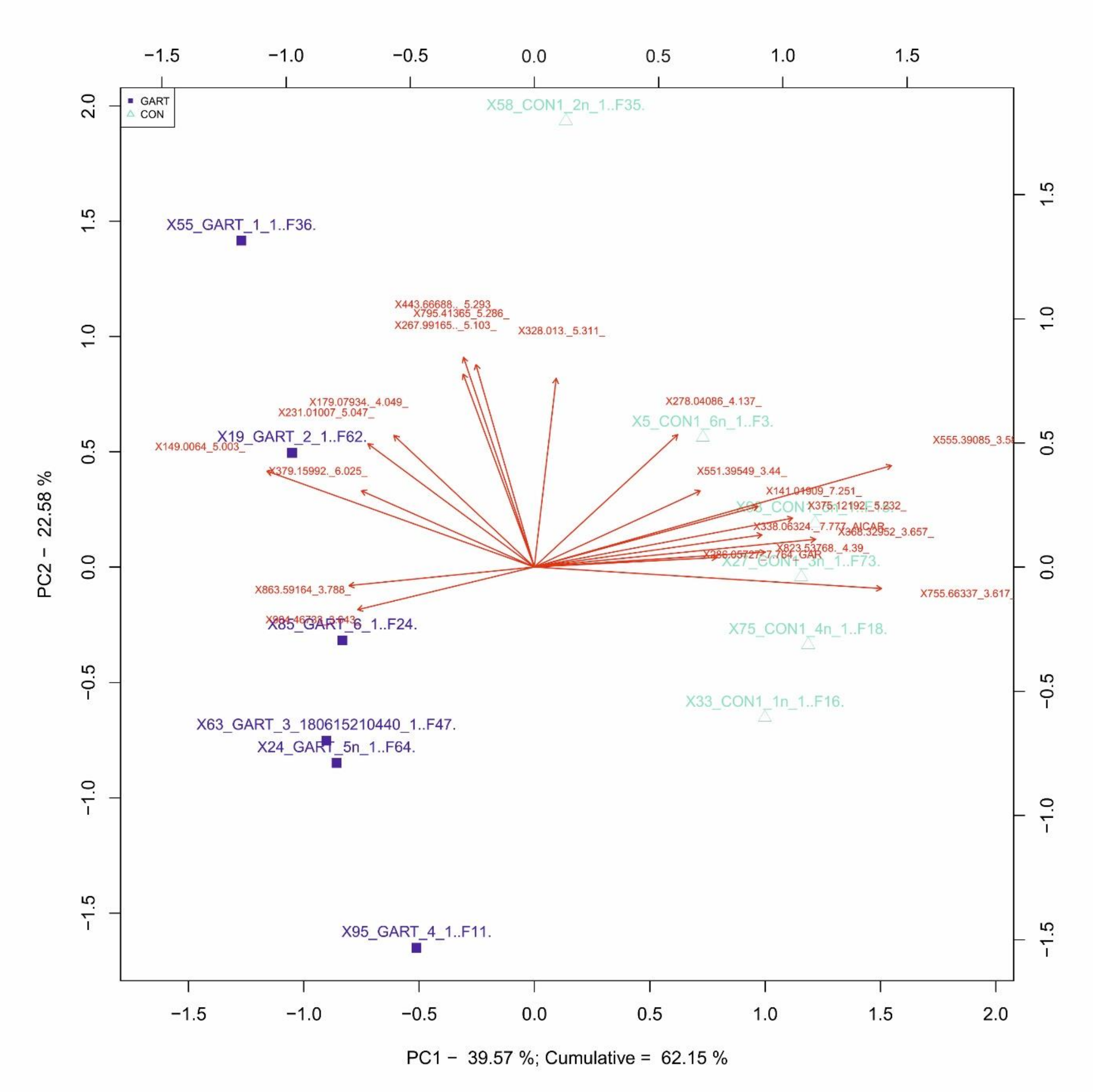

Figure S6: PCA biplots of GART-deficient cells compared to control group subjected to an untargeted analysis. The length and direction of arrows in the figure are given by loadings of metabolites of the first two components. Name of each sample contains information about batch order, cell line and identification numbers assigned automatically by Compound Discoverer.

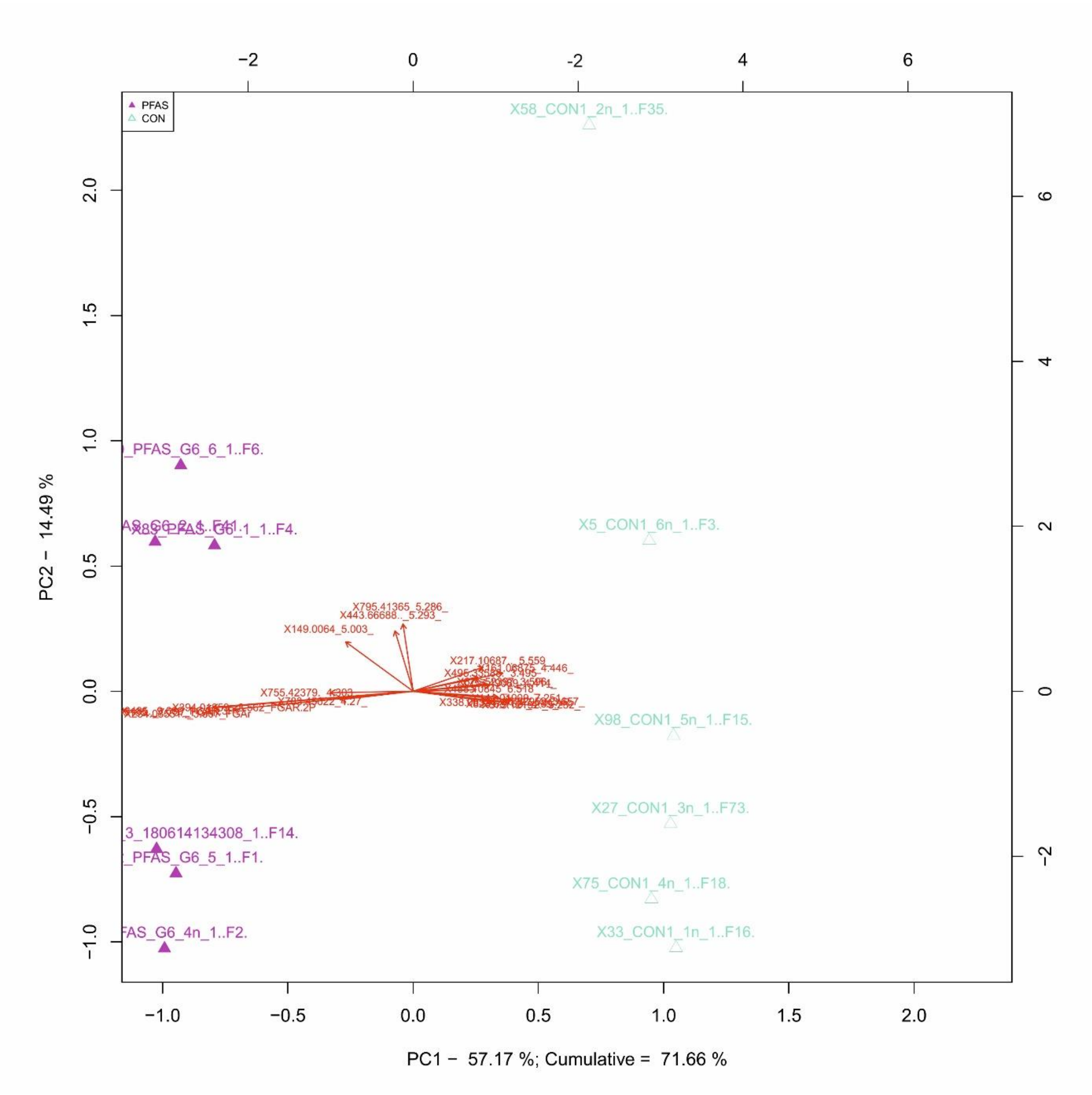

Figure S7: PCA biplots of PFAS-deficient cells compared to control group subjected to an untargeted analysis. The length and direction of arrows in the figure are given by loadings of metabolites of the first two components. Name of each sample contains information about batch order, cell line and identification numbers assigned automatically by Compound Discoverer.

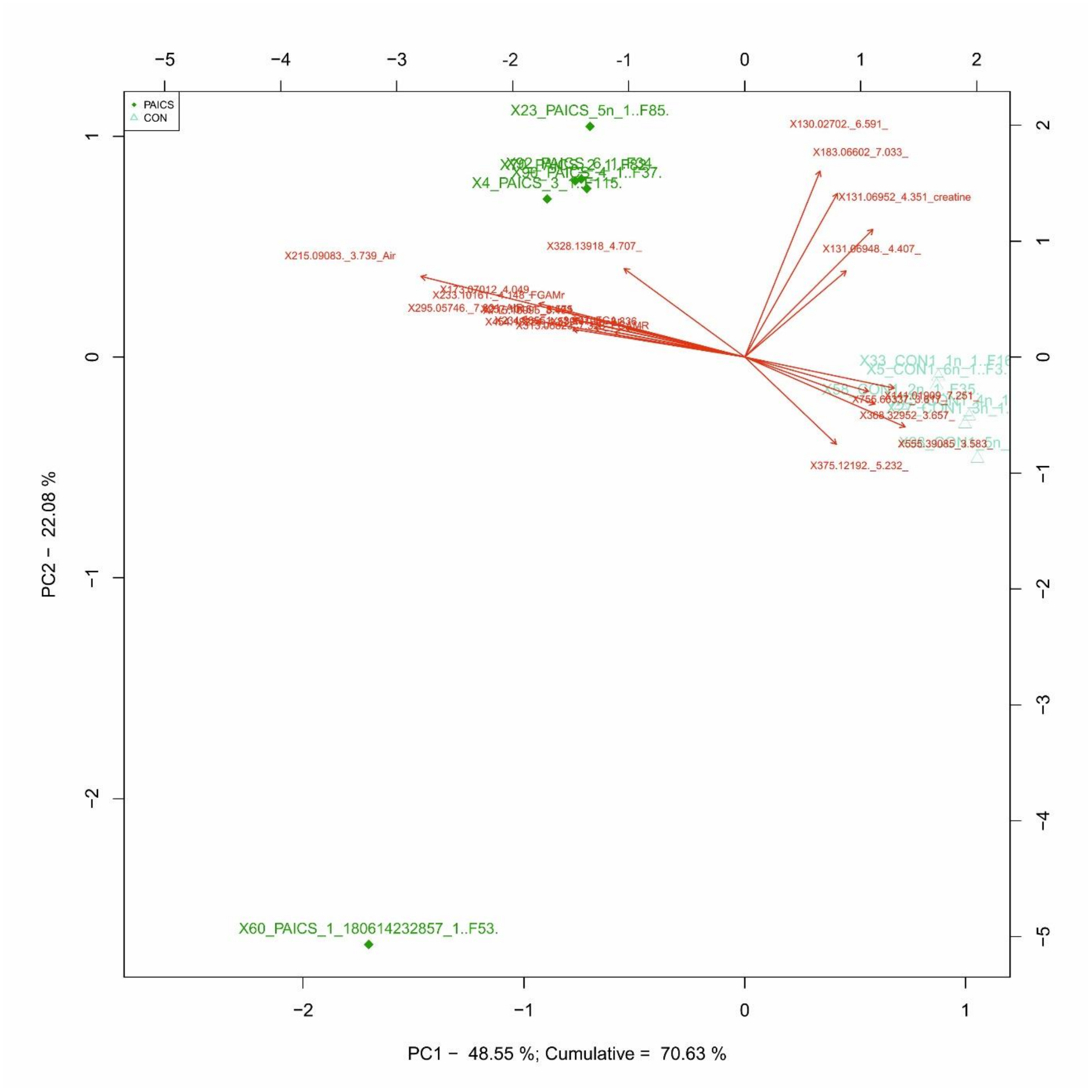

Figure S8: PCA biplots of PAICS-deficient cells compared to control group subjected to an untargeted analysis. The length and direction of arrows in the figure are given by loadings of metabolites of the first two components. Name of each sample contains information about batch order, cell line and identification numbers assigned automatically by Compound Discoverer.

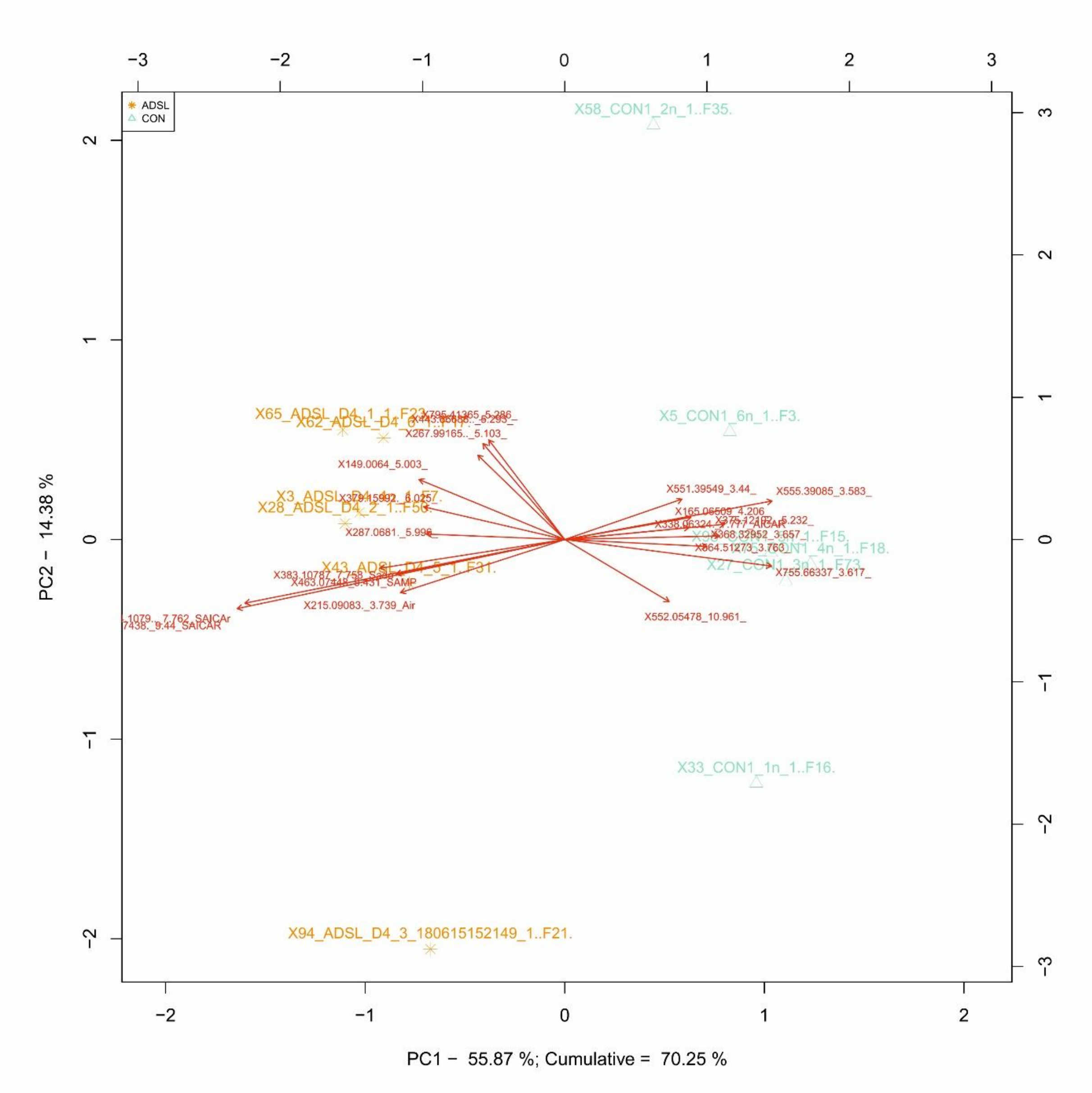

Figure S9: PCA biplots of ADSL-deficient cells compared to control group subjected to an untargeted analysis. The length and direction of arrows in the figure are given by loadings of metabolites of the first two components. Name of each sample contains information about batch order, cell line and identification numbers assigned automatically by Compound Discoverer.

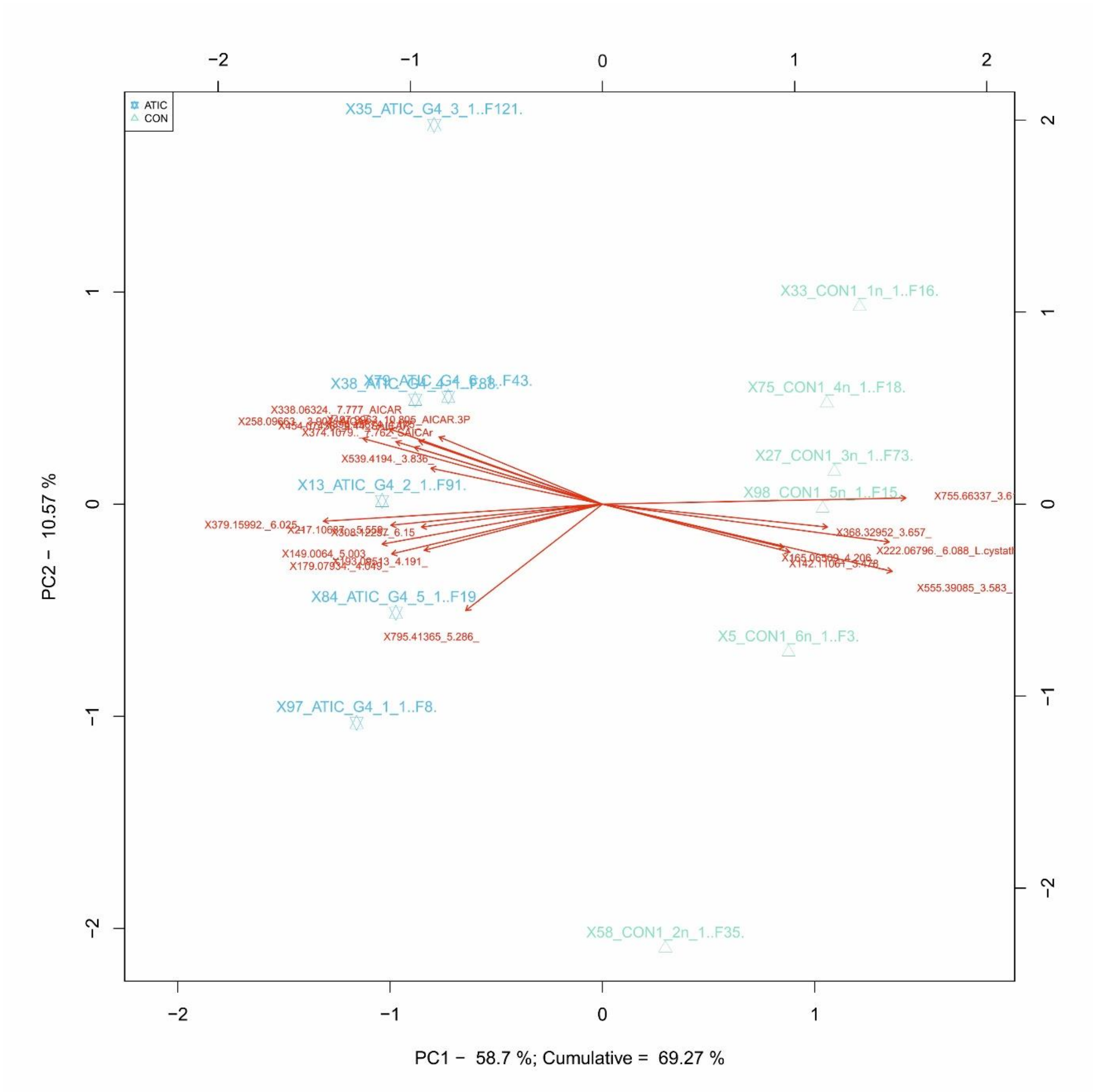

Figure S10: PCA biplots of ATIC-deficient cells compared to control group subjected to an untargeted analysis. The length and direction of arrows in the figure are given by loadings of metabolites of the first two components. Name of each sample contains information about batch order, cell line and identification numbers assigned automatically by Compound Discoverer.

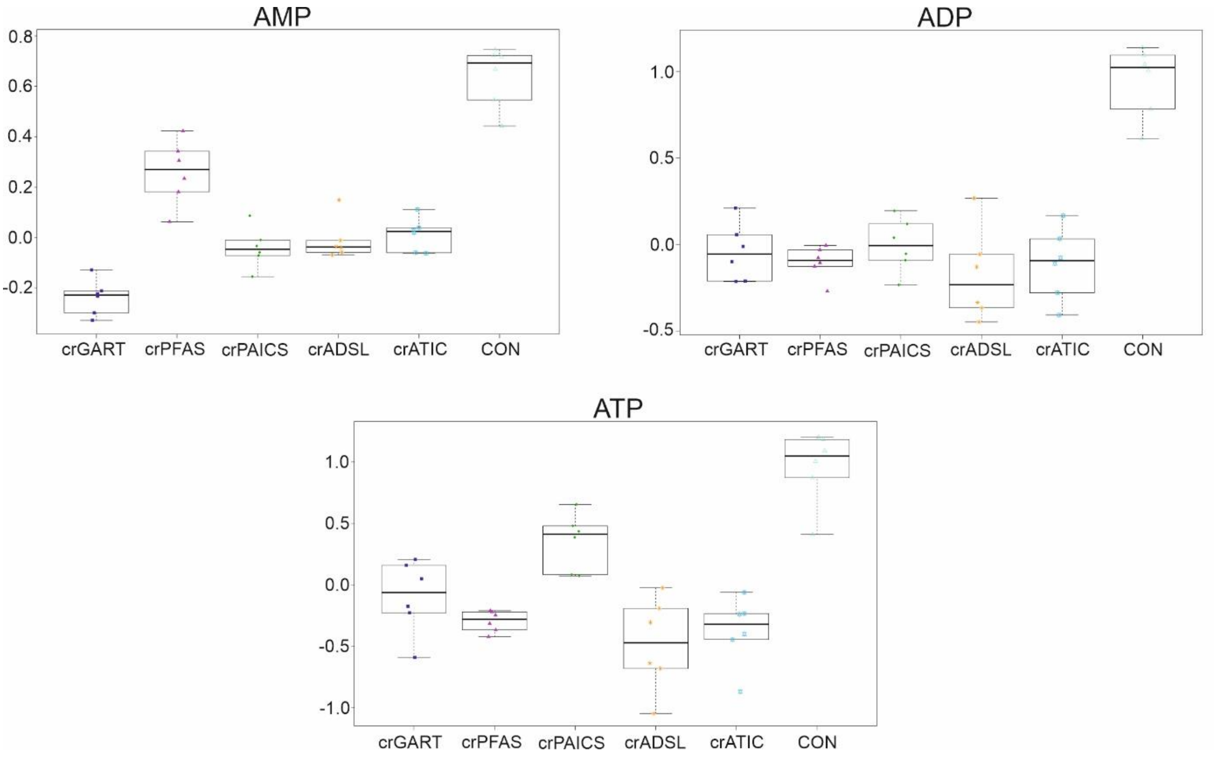

Figure S11: Boxplots of adenosine nucleotides in PDNS deficient cell lines and control cells: adenosine monophosphate (AMP), adenosine diphosphate (ADP), and adenosine triphosphate (ATP).

Table S7: Significant metabolites of VIP-OPLS-DA (mean VIP higher than 1) detected in crGART cells compared to control in targeted analysis.

order of significance	metabolite	mean VIP	SD VIP	p1	fold change	p-value
1	phosphoserine	3.25	0.07	8.06	3.80	4.60E-07
2	ethanolaminephosphate	2.35	0.08	-5.92	-1.99	1.06E-04
3	serine	2.25	0.08	5.54	2.06	3.25E-05
4	glucose/galactose-1-phosphate / glucose/fructose-6-phosphate	2.03	0.03	-5.10	-1.47	2.86E-08
5	N-acetylputrescine	1.96	0.13	5.03	1.73	1.11E-03
6	glycerate-3-phosphate	1.91	0.06	4.68	1.35	1.10E-05
7	2-hydroxyisovalerate	1.85	0.06	-4.62	-1.22	1.47E-04
8	docosenoylcarnitine	1.84	0.21	4.38	1.64	1.37E-02
9	deoxyadenosine triphosphate	1.84	0.04	-4.70	-1.24	5.69E-06
10	adenylylsulphate	1.79	0.04	-4.52	-1.15	4.08E-06
11	guanosine monophosphate	1.76	0.04	-4.42	-1.19	1.09E-06
12	palmitoylcarnitine	1.73	0.09	4.26	-1.19	1.73E-04
13	myristoylcarnitine	1.69	0.09	4.13	1.15	2.03E-04
14	palmitoleoylcarnitine	1.67	0.07	4.09	1.00	1.36E-05
15	3-hydroxy-tetradecenoyl carnitine	1.66	0.06	4.16	1.06	4.05E-05
16	guanidinoacetate	1.65	0.13	-3.96	-1.31	3.92E-03

17	adenosine triphosphate	1.62	0.07	-4.19	-1.11	1.07E-04
18	adenosine diphosphate	1.62	0.04	-4.14	-1.08	4.62E-06
19	3-hydroxy-palmitoleoylcarnitine	1.61	0.09	3.98	1.17	4.31E-04
20	guanosine	1.61	0.10	-4.04	-1.00	1.53E-02
21	guanosine diphosphate	1.61	0.11	-4.22	-1.25	8.34E-04
22	guanosine diphosphate mannose	1.60	0.06	-4.04	-0.97	3.75E-04
23	adenosine	1.59	0.06	-4.08	-1.11	6.54E-04
24	glutamic acid	1.59	0.04	3.93	0.96	7.81E-07
25	dihydrofolate	1.58	0.08	-3.94	-0.95	1.58E-03
26	hydroxyproline	1.57	0.07	-3.81	-0.96	2.54E-05
27	oleoylcarnitine	1.56	0.11	3.81	1.08	1.52E-03
28	adenosine monophosphate	1.56	0.02	-3.94	-0.92	2.85E-07
29	guanosine diphosphate fucose	1.50	0.04	-3.76	-0.80	8.16E-06
30	4-aminobutanoate	1.49	0.05	3.73	0.85	4.74E-06
31	acetylmethionine	1.49	0.05	-3.76	-0.86	4.26E-05
32	tiglylcarnitine	1.47	0.08	3.57	0.73	1.63E-03
33	glycerol-3-phosphate	1.44	0.06	-3.61	-0.89	1.11E-04
34	phosphocreatine	1.39	0.08	3.36	0.72	5.11E-04
35	urea	1.38	0.12	-3.44	-1.12	9.64E-03
36	taurine	1.38	0.04	3.41	0.66	6.40E-06
37	linoleylcarnitine	1.36	0.11	3.42	0.74	3.04E-03
38	valerylcarnitine	1.31	0.07	3.24	0.50	1.15E-03
39	aspartic acid	1.30	0.05	3.17	0.70	3.00E-05
40	carnitine	1.30	0.04	3.18	0.58	9.20E-06
41	cytidine	1.29	0.11	-3.11	-0.83	8.72E-03
42	galactitol/manitol	1.29	0.10	3.29	0.78	4.12E-03
43	phosphoenolpyruvate	1.28	0.11	3.22	0.66	2.45E-02
44	lauroylcarnitine	1.26	0.08	3.13	0.57	2.92E-03
45	propionylcarnitine	1.26	0.03	3.13	0.57	1.89E-05
46	fucose	1.25	0.21	3.31	0.72	6.97E-02
47	gluconate	1.24	0.11	3.25	0.87	3.85E-03
48	deoxyadenosine monophosphate	1.23	0.06	-3.06	-0.56	1.81E-03
49	pseudouridine	1.22	0.07	-2.95	-0.58	1.85E-04
50	sorbitol-6-phosphate	1.18	0.13	-3.00	-0.89	1.53E-02
51	arabitol/ribitol	1.18	0.10	-2.81	-0.69	5.31E-03
52	stearoylcarnitine	1.17	0.10	2.84	0.64	4.19E-03
53	glucosamine-6-phosphate	1.16	0.14	-2.86	-0.71	1.56E-02
54	UDP-N-acetyl-D-glucosamine	1.11	0.07	-2.83	-0.63	7.41E-04
55	S-lactoylglutathione	1.10	0.04	2.76	0.52	1.17E-02
56	glucose/fructose/galactose/mannose	1.10	0.14	2.98	0.70	1.93E-02
57	oxalic acid	1.08	0.15	2.84	0.74	2.54E-02
58	betaine	1.07	0.04	2.68	0.47	2.22E-04
59	xanthosine	1.01	0.18	-2.49	-0.56	4.11E-02
60	cytidine diphosphate	1.01	0.11	-2.63	-0.66	1.07E-02
61	proline	1.01	0.08	-2.52	-0.52	2.77E-03

Table S8: Significant metabolites of VIP-OPLS-DA (mean VIP higher than 1) detected in crPFAS cells compared to control in targeted analysis.

	metabolite			p1		p-value

order of significance		mean VIP	SD VIP		fold change	
1	docosenoylcarnitine	2.41	0.08	8.36	2.83	5.35E-04
2	phosphoserine	2.39	0.04	8.29	2.98	5.17E-08
3	ethanolaminephosphate	2.07	0.05	-7.10	-2.03	1.74E-04
4	oleoylcarnitine	2.06	0.05	7.15	2.21	6.00E-05
5	palmitoylcarnitine	2.06	0.04	7.12	2.25	1.05E-05
6	3-hydroxy-tetradecenoyl carnitine	2.02	0.04	6.89	1.89	1.64E-09
7	3-hydroxy-palmitoleoylcarnitine	1.97	0.04	6.80	1.99	2.76E-05
8	cytidine diphosphate choline	1.95	0.07	-6.89	-2.07	7.32E-05
9	palmitoleoylcarnitine	1.93	0.03	6.65	1.76	9.15E-09
10	eicosenoylcarnitine	1.89	0.05	6.60	1.82	7.34E-06
11	linoleylcarnitine	1.87	0.05	6.39	1.58	5.65E-05
12	acetylmethionine	1.87	0.05	-6.33	-1.75	1.97E-06
13	hypoxanthine	1.80	0.24	-6.35	-2.94	1.03E-02
14	myristoylcarnitine	1.77	0.04	6.13	1.61	1.88E-05
15	phosphoenolpyruvate	1.76	0.06	6.20	1.51	6.29E-04
16	glycerate-3-phosphate	1.73	0.03	6.03	1.49	2.10E-06
17	stearoylcarnitine	1.68	0.04	5.83	1.44	2.98E-05
18	propionylcarnitine	1.64	0.02	5.66	1.37	6.08E-07
19	glucose/galactose-1-phosphate / glucose/fructose-6-phosphate	1.63	0.03	-5.53	-1.34	1.74E-08
20	betaine	1.62	0.03	5.55	1.35	7.79E-09
21	deoxyadenosine triphosphate	1.62	0.03	-5.48	-1.34	2.96E-05
22	adenosine triphosphate	1.60	0.04	-5.35	-1.33	6.88E-05
23	adenylylsulphate	1.59	0.02	-5.45	-1.26	8.82E-09
24	glucosaminephosphate	1.58	0.06	-5.44	-1.41	1.19E-04
25	acetylcarnitine	1.58	0.03	5.47	1.30	2.26E-07
26	panthotenate	1.57	0.08	-5.18	-1.39	3.49E-04
27	cystathionine	1.51	0.05	-5.00	-1.08	7.90E-06
28	serine	1.51	0.06	5.27	1.20	1.05E-04
29	galactitol/manitol	1.50	0.04	5.17	1.04	5.84E-05
30	adenosine diphosphate	1.46	0.03	-4.98	-1.11	9.11E-06
31	guanidinoacetate	1.46	0.04	5.03	1.08	2.44E-05
32	4-aminobutanoate	1.39	0.04	4.81	1.02	2.65E-06
33	valerylcarnitine	1.38	0.03	4.85	1.00	2.03E-06
34	butyrylcarnitine	1.37	0.04	4.74	1.04	3.88E-06
35	carnitine	1.33	0.02	4.67	0.91	2.60E-07
36	gunidinobutanoate	1.33	0.03	4.66	0.97	2.17E-06
37	melibiose/sucrose/lactose/maltose	1.32	0.22	-4.75	-1.84	2.86E-02
38	2-hydroxyisovalerate	1.31	0.07	-4.44	-0.85	7.62E-04
39	urea	1.31	0.15	-4.72	-1.47	8.50E-03
40	UDP-N-acetyl-D-glucosamine	1.31	0.04	-4.37	-0.87	3.86E-06
41	oxalic acid	1.30	0.11	4.32	1.17	2.99E-03
42	sorbitol-6-phosphate	1.29	0.03	-4.36	-0.82	2.82E-08
43	fucose	1.26	0.17	4.19	0.91	4.37E-02
44	cytidine	1.26	0.03	-4.33	-0.77	5.03E-06
45	S-adenosyl-L-methionine	1.25	0.04	-4.25	-0.85	2.46E-06

46	malate	1.18	0.02	-4.04	-0.68	1.05E-08
47	xanthosine	1.17	0.08	-4.10	-0.69	2.52E-03
48	asparagine	1.15	0.12	4.30	0.85	1.11E-02
49	guanosine diphosphate mannose	1.13	0.03	-3.84	-0.67	1.20E-06
50	glutamic acid	1.12	0.03	3.95	0.68	8.07E-06
51	5'-methylthioadenosine	1.11	0.05	-3.87	-0.64	1.40E-04
52	nicotinate	1.11	0.11	-4.08	-0.86	1.30E-02
53	fumarate/caproic acid/3-methyl-2-oxobutanoate	1.08	0.02	-3.73	-0.57	2.45E-07
54	dihydrofolate	1.08	0.09	-3.73	-0.72	2.35E-03
55	aconitate	1.07	0.04	3.82	0.54	4.34E-04
56	threonate	1.06	0.06	-3.61	-0.63	3.08E-04
57	deoxyadenosine monophosphate	1.06	0.06	-3.55	-0.58	1.61E-03
58	xanthine	1.04	0.05	-3.71	-0.48	7.99E-04
59	inosine monophosphate	1.02	0.08	3.57	0.69	2.39E-03
60	N-acetylserotonine	1.02	0.17	-3.81	-0.99	4.83E-02
61	2-aminoadipate	1.01	0.05	3.50	0.53	9.47E-05
62	arachidonic acid	1.01	0.15	-3.60	-0.93	2.61E-02
63	N-acetylglucosamine-6-phosphate	1.00	0.09	-3.50	-0.63	1.40E-02

Table S9: Significant metabolites of VIP-OPLS-DA (mean VIP higher than 1) detected in crPAICS cells compared to control in targeted analysis.

order of significance	metabolite	mean VIP	SD VIP	p1	fold change	p-value
1	propenoylcarnitine	3.36	0.09	8.84	4.50	1.38E-06
2	phosphoserine	3.07	0.06	8.12	3.79	7.41E-09
3	ethanolaminephosphate	2.71	0.06	-7.24	-2.69	4.01E-05
4	glycerate-3-phosphate	1.99	0.05	5.22	1.54	1.76E-06
5	dihydrofolate	1.93	0.06	-5.14	-1.41	5.61E-06
6	fucose	1.81	0.13	4.96	1.30	1.13E-02
7	galactitol/manitol	1.81	0.04	4.83	1.18	4.17E-07
8	cytidine diphosphate choline	1.79	0.10	-4.81	-1.31	7.38E-04
9	serine	1.78	0.07	4.70	1.40	1.37E-04
10	guanidinoacetate	1.76	0.15	-4.49	-1.53	4.33E-03
11	glycerol-3-phosphate	1.74	0.04	-4.64	-1.21	1.65E-05
12	propionylcarnitine	1.72	0.04	4.54	1.19	2.12E-07
13	docosenoylcarnitine	1.71	0.16	4.43	1.31	1.67E-02
14	phosphoenolpyruvate	1.69	0.08	4.47	1.02	3.62E-03
15	glucose/galactose-1-phosphate / glucose/fructose-6-phosphate	1.69	0.03	-4.56	-1.08	2.55E-06
16	cystathionine	1.68	0.04	-4.52	-1.14	1.92E-07
17	guanosine diphosphate	1.66	0.08	-4.63	-1.24	4.03E-04
18	adenosine	1.62	0.06	-4.43	-1.13	6.81E-04
19	cytidine	1.62	0.05	-4.33	-1.02	5.92E-04
20	urea	1.58	0.09	-4.21	-1.22	3.05E-03

21	valerylcarnitine	1.56	0.04	4.10	0.89	4.91E-07
22	butyrylcarnitine	1.56	0.04	4.16	0.99	1.77E-05
23	adenosine diphosphate	1.54	0.03	-4.22	-1.03	6.54E-06
24	carnitine	1.53	0.05	3.95	0.89	3.07E-07
25	2-hydroxyisovalerate	1.49	0.11	-3.89	-0.87	5.64E-03
26	guanosine diphosphate mannose	1.49	0.04	-4.05	-1.22	5.51E-04
27	glutamic acid	1.46	0.03	3.85	0.84	1.51E-06
28	5-aminolevulinate	1.40	0.03	-3.75	-0.72	7.34E-07
29	N-acetylputrescine	1.38	0.10	3.85	0.81	7.92E-03
30	guanosine monophosphate	1.37	0.02	-3.67	-0.74	4.69E-06
31	adenylylsulphate	1.37	0.06	-3.73	-0.72	1.90E-03
32	glutathione (oxidized)	1.37	0.05	3.60	0.81	4.41E-05
33	guanosine	1.36	0.08	-3.79	-0.77	2.49E-03
34	4-hydroxybenzaldehyde	1.34	0.07	-3.54	-0.73	6.95E-04
35	adenosine monophosphate	1.32	0.02	-3.59	-0.74	1.45E-06
36	hexanoylcarnitine	1.31	0.05	3.56	0.79	5.02E-04
37	UDP-N-acetyl-D-glucosamine	1.28	0.03	-3.51	-0.65	1.57E-05
38	aspartic acid	1.27	0.04	3.32	0.64	3.74E-05
39	tiglylcarnitine	1.26	0.08	3.27	0.63	6.14E-03
40	hydroxyproline	1.25	0.06	-3.18	0.18	1.58E-04
41	leucylleucine	1.24	0.11	3.25	0.49	6.79E-03
42	taurine	1.24	0.04	3.28	0.55	2.23E-05
43	cytidine diphosphate	1.21	0.09	-3.42	-0.67	3.92E-03
44	5'-methylthioadenosine	1.20	0.07	-3.08	-0.63	4.48E-04
45	deoxyadenosine triphosphate	1.16	0.08	-3.28	-0.70	1.08E-03
46	uridine	1.16	0.19	-3.12	-1.06	3.81E-02
47	3-hydroxy-palmitoleoylcarnitine	1.14	0.11	-3.13	-0.69	1.04E-02
48	deoxyadenosine monophosphate	1.11	0.11	-3.02	-0.63	5.09E-03
49	glucose/fructose/galactose/mannose	1.11	0.13	3.20	0.70	1.75E-02
50	adenosine triphosphate	1.10	0.09	-3.16	-0.64	2.95E-03
51	homoarginine	1.10	0.12	2.75	0.74	5.92E-03
52	2-aminoadipate	1.08	0.06	-2.86	-0.50	2.25E-04
53	glucosminephosphate	1.07	0.14	-3.04	-0.55	2.35E-02
54	gluconate	1.07	0.10	3.02	0.65	1.07E-02
55	hydrohyglutarate	1.06	0.05	2.87	0.48	1.29E-03
56	sorbitol-6-phosphate	1.05	0.06	-2.87	-0.56	3.06E-03
57	N-acetylserotonine	1.03	0.18	-2.79	-0.61	7.49E-02
58	uridine diphosphate glucuronate	1.03	0.06	2.63	0.49	3.81E-04
59	eicosenoylcarnitine	1.03	0.14	-2.88	-0.67	1.57E-02
60	glutathione (reduced)	1.03	0.06	2.84	0.50	9.18E-04
61	xanthine	1.01	0.10	-2.56	-0.42	9.17E-03
62	hydroxycinnamate	1.01	0.12	-2.68	-0.63	2.05E-02
63	methionine	1.01	0.03	2.76	0.43	4.90E-05

Table S10: Significant metabolites of VIP-OPLS-DA (mean VIP higher than 1) detected in crADSL cells compared to control in targeted analysis.

order of significance	metabolite	mean VIP	SD VIP	p1	fold change	p-value
1	docosenoylcarnitine	2.65	0.10	8.21	3.01	1.78E-04
2	oleoylcarnitine	2.25	0.06	7.05	2.45	7.86E-06
3	eicosenoylcarnitine	2.18	0.07	6.78	2.16	2.22E-06
4	linoleylcarnitine	2.10	0.05	6.63	1.89	3.17E-06
5	phosphoserine	2.00	0.07	6.16	2.09	2.19E-06
6	palmitoleoylcarnitine	1.95	0.05	6.16	1.75	5.20E-07
7	stearoylcarnitine	1.87	0.05	5.83	1.58	2.35E-06
8	palmitoylcarnitine	1.84	0.06	5.77	1.68	1.93E-05
9	galactitol/manitol	1.83	0.04	5.82	1.39	1.54E-06
10	deoxyadenosine triphosphate	1.82	0.03	-5.92	-1.53	4.55E-06
11	adenosine triphosphate	1.72	0.04	-5.68	-1.52	3.21E-05
12	2-hydroxyisovalerate	1.72	0.09	-5.35	-1.20	5.24E-04
13	fucose	1.67	0.14	5.51	1.23	1.05E-02
14	acetylmethionine	1.67	0.05	-5.32	-1.34	7.52E-06
15	UDP-N-acetyl-D-glucosamine	1.65	0.02	-5.27	-1.28	1.48E-08
16	glucose/galactose-1-phosphate / glucose/fructose-6-phosphate	1.65	0.03	-5.21	-1.26	1.80E-07
17	deoxythymidine triphosphate	1.63	0.03	-5.33	-1.30	1.50E-05
18	cystathionine	1.63	0.02	-5.23	-1.21	3.44E-07
19	cytidine triphosphate	1.62	0.06	-5.38	-1.40	2.04E-04
20	cytidine diphosphate	1.61	0.04	-5.22	-1.29	3.59E-05
21	3-hydroxy-tetradecenoyl carnitine	1.58	0.05	5.00	1.22	4.72E-06
22	carnitine	1.58	0.04	4.93	1.09	2.95E-08
23	3-hydroxy-palmitoleoylcarnitine	1.56	0.07	4.86	1.25	2.16E-04
24	uridine triphosphate	1.56	0.07	-5.19	-1.31	4.03E-04
25	valerylcarnitine	1.54	0.04	4.87	1.10	4.05E-07
26	adenosine diphosphate	1.53	0.03	-5.01	-1.26	1.33E-05
27	creatine/5-aminolevulinate	1.51	0.03	-4.74	-1.02	1.54E-07
28	uridine diphosphate	1.51	0.03	-4.90	-1.05	4.94E-05
29	glycerate-3-phosphate	1.45	0.07	4.44	1.04	4.89E-05
30	lactate	1.42	0.06	-4.32	-0.94	1.25E-05
31	deoxyadenosine monophosphate	1.40	0.02	-4.51	-0.88	6.32E-07
32	glycerol-3-phosphate	1.35	0.04	-4.30	-0.89	4.12E-05
33	proline	1.32	0.04	-4.08	-0.77	1.26E-07
34	guanosine diphosphate mannose	1.31	0.04	-4.14	-0.79	1.11E-05
35	serine	1.29	0.09	3.93	0.77	2.52E-03
36	acetylcarnitine	1.27	0.04	-3.98	-0.71	1.46E-06
37	glucose/fructose/galactose/mannose	1.27	0.09	4.28	0.94	3.46E-03
38	aspartic acid	1.25	0.04	3.85	0.73	1.01E-05
39	propionylcarnitine	1.25	0.03	3.95	0.76	6.84E-06
40	pseudouridine	1.24	0.07	-3.69	-0.71	2.96E-04
41	gluconate	1.24	0.08	4.00	0.92	1.40E-03
42	lauroylcarnitine	1.24	0.08	-3.89	-0.89	8.51E-04
43	dihydrofolate	1.24	0.18	-3.59	-1.20	3.43E-02
44	hydroxyproline	1.23	0.06	-3.71	-0.65	6.43E-05

45	butyrylcarnitine	1.22	0.05	-3.80	-0.72	6.43E-05
46	adenine	1.21	0.12	3.95	1.05	1.40E-02
47	hexanoylcarnitine	1.20	0.06	-3.67	-0.67	3.97E-04
48	adenosine monophosphate	1.18	0.02	-3.83	-0.73	1.99E-06
49	adenylylsulphate	1.16	0.11	-3.78	-0.97	6.13E-03
50	guanosine diphosphate	1.16	0.11	-3.90	-0.90	5.38E-03
51	decanoylcarnitine	1.13	0.14	-3.27	-0.89	1.62E-02
52	glucuronate	1.12	0.09	3.54	0.75	2.32E-03
53	uridine	1.12	0.19	-3.32	-1.24	2.87E-02
54	choline	1.08	0.03	3.54	0.64	2.97E-05
55	ribose	1.07	0.09	3.63	0.67	4.63E-03
56	panthotenate	1.07	0.15	-3.77	-1.00	2.24E-02
57	glucosaminephosphate	1.06	0.11	-3.59	-0.71	1.22E-02
58	xanthine	1.06	0.09	-3.07	-0.54	3.50E-03
59	phosphoenolpyruvate	1.06	0.10	3.31	0.57	3.50E-02
60	threonate	1.06	0.06	-3.35	-0.56	1.16E-03
61	betaine	1.05	0.02	3.34	0.55	9.33E-05
62	succinate/methylmalonate	1.04	0.06	3.48	0.54	1.10E-03
63	glutamic acid	1.04	0.04	3.23	0.53	5.34E-05
64	taurine	1.04	0.05	3.22	0.51	1.11E-04
65	tiglylcarnitine	1.02	0.09	3.16	0.60	1.14E-02
66	leucylleucine	1.02	0.16	3.23	0.32	1.19E-01

Table S11: Significant metabolites of VIP-OPLS-DA (mean VIP higher than 1) detected in crATIC cells compared to control in targeted analysis.

order of significance	metabolite	mean VIP	SD VIP	p1	fold change	p-value
1	cystahionine	2.84	0.05	-10.03	-4.05	3.44E-07
2	phosphoserine	2.44	0.08	8.37	3.36	2.19E-06
3	guanidinoacetate	1.94	0.04	6.93	1.87	2.66E-02
4	guanosine diphosphate	1.79	0.06	-6.71	-1.82	5.38E-03
5	galactitol/manitol	1.77	0.04	6.32	1.47	1.54E-06
6	deoxyadenosine triphosphate	1.74	0.03	-6.37	-1.55	4.55E-06
7	aspartate	1.73	0.04	6.02	1.52	1.01E-05
8	panthotenate	1.72	0.04	-6.43	-1.63	2.24E-02
9	propionylcarnitine	1.70	0.04	6.01	1.44	6.84E-06
10	butyrylcarnitine	1.70	0.04	6.03	1.50	6.43E-05
11	guanosine diphosphate mannose	1.69	0.05	-5.95	-1.53	1.11E-05
12	acetylmethionine	1.68	0.05	-6.04	-1.40	7.52E-06
13	N-acetylgalactosamine/N-acetylglucosamin/N-acetylmannosamine	1.64	0.06	5.93	1.45	3.79E-02
14	carnitine	1.62	0.04	5.59	1.28	2.95E-08
15	glucose/galactose-1-phosphate / glucose/fructose-6-phosphate	1.59	0.03	-5.64	-1.27	1.80E-07
16	spermine	1.59	0.03	5.70	1.39	2.27E-03
17	adenosinetriphosphate	1.58	0.02	-5.90	-1.37	3.21E-05
18	ethanolaminephosphate	1.57	0.07	-5.59	-1.18	3.72E-01

19	guanosine monophosphate	1.57	0.03	-5.50	-1.24	8.20E-01
20	betaine	1.56	0.03	5.58	1.22	9.33E-05
21	uridine diphosphate-N-acetylglucosamine	1.54	0.05	5.30	1.15	1.48E-08
22	fucose	1.54	0.12	5.87	1.30	1.05E-02
23	adenylylsulphate	1.53	0.03	-5.41	-1.19	6.13E-03
24	valerylcarnitine	1.49	0.04	5.23	1.10	4.05E-07
25	phosphoenolpyruvate	1.49	0.08	5.13	1.11	3.50E-02
26	melibiose/sucrose/lactose/maltose	1.49	0.11	-5.25	-1.70	2.94E-01
27	glycerate-3-phosphate	1.49	0.07	4.98	1.26	4.89E-05
28	glucose/fructose/galactose/mannose	1.47	0.05	5.56	1.28	3.46E-03
29	b-alanine/alanine/sarcosine	1.43	0.04	-4.97	-1.11	8.45E-01
30	adenosine diphosphate	1.42	0.02	-5.25	-1.12	1.33E-05
31	glucosaminephosphate	1.41	0.08	-5.16	-1.18	1.22E-02
32	ribose	1.40	0.04	5.22	1.06	4.63E-03
33	acetylcarnitine	1.39	0.04	4.82	1.01	1.46E-06
34	N-acetylglucosamine-6-phosphate	1.35	0.08	4.62	0.95	1.08E-01
35	2-hydroxyisovalerate	1.34	0.10	-4.57	-1.11	5.24E-04
36	gluconate	1.34	0.05	5.02	1.11	1.40E-03
37	glucosamine	1.34	0.05	5.08	1.13	2.48E-02
38	guanidinobutanoate	1.32	0.04	4.60	0.93	1.40E-02
39	docosenoylcarnitine	1.31	0.17	4.15	0.88	1.78E-04
40	lactate	1.29	0.06	-4.31	-0.88	1.25E-05
41	tiglylcarnitine	1.26	0.07	4.21	0.88	1.14E-02
42	homoarginine	1.25	0.05	-4.69	-0.69	2.83E-01
43	thiamine	1.24	0.09	4.92	1.20	6.22E-02
44	glutamic acid	1.23	0.03	4.30	0.80	5.34E-05
45	deoxyadenosine monophosphate	1.22	0.04	-4.35	-0.79	6.32E-07
46	dihydrofolate	1.19	0.13	-4.00	-0.93	3.43E-02
47	glycerol-3-phosphate	1.18	0.05	-4.18	-0.76	4.12E-05
48	urea	1.16	0.12	-4.15	-1.14	3.25E-01
49	cytidine diphosphate choline	1.15	0.14	-4.06	-0.90	1.35E-01
50	aspartate	1.14	0.10	-4.63	-1.06	7.38E-02
51	sorbitol-6-phosphate	1.13	0.05	-4.06	-0.78	6.73E-02
52	3-hydroxy-palmitoleoylcarnitine	1.12	0.09	-4.04	-0.82	2.16E-04
53	taurine	1.12	0.04	-3.87	-0.69	1.11E-04
54	S-adenosylmethionine	1.11	0.05	-3.81	-0.64	4.06E-03
55	adenosine monophosphate	1.11	0.02	-4.01	-0.67	1.99E-06
56	threonate	1.11	0.05	-3.91	-0.64	1.16E-03
57	proline	1.08	0.04	-3.72	-0.62	1.26E-07
58	phosphocreatine	1.08	0.06	3.59	0.62	1.75E-01
59	cytidine monophosphate	1.08	0.04	3.71	0.58	5.24E-02
60	erythrose-4-phosphate	1.07	0.04	-3.73	-0.61	4.92E-02
61	xanthosine	1.07	0.11	-3.49	-0.50	3.95E-02
62	guanosine diphosphate fucose	1.05	0.03	-3.72	-0.50	1.82E-05
63	serine	1.04	0.10	3.48	0.78	2.52E-03
64	guanosine	1.04	0.08	-3.82	-0.68	2.98E-01
65	myristoylcarnitine	1.02	0.06	-3.84	-0.58	4.29E-01
66	CMP-N-acetylneuraminate	1.02	0.07	3.53	0.66	4.57E-03
67	ribose-5-phosphate/xylose-5-phosphate	1.02	0.05	-3.47	-0.50	7.71E-03
68	myoinositol	1.00	0.07	4.00	0.69	5.52E-01
69	allantoin	1.00	0.09	4.06	0.67	4.61E-01

Table S12: List of abbreviations.

Abbreviation	Metabolite
acetyl-CoA	acetyl-coenzymeA
adenosylHcys	S-adenosylhomocysteine
adenosylMet	S-adenosylmethionine
ADP	adenosine diphosphate
AMP	adenosine monophosphate
ATP	adenosine triphosphate
C0	carnitine
C10	decanoylcarnitine
C12	lauroylcarnitine
C14	myristoylcarnitine
C14-1OH	3-hydroxy-tetradecenoyl carnitine
C16-1	palmitoleoylcarnitine
C16-1OH	3-hydroxy-palmitoleoylcarnitine
C18	stearoylcarnitine
C18-1	oleoylcarnitine
C18-2	linoleylcarnitine
C2	acetylcarnitine
C20-1	eicosenoylcarnitine
C22-1	docosenoylcarnitine
C3	propionylcarnitine
C3-1	propenoylcarnitine
C4	butyrylcarnitine
C5	valerylcarnitine
C5-1	tiglylcarnitine
C6	hexanoylcarnitine
C8	octanoylcarnitine
CDP	cytidine diphosphate
CMP	cytidine monophosphate
CTP	cytidine triphosphate
erythrose4P	erythrose-4-phosphate
fum/cap/3M2Obut	fumarate/caproate/3-methyl-3-hydroxybutanoate
GDP	guanosine diphosphate
glucosamineP	glucosamine phosphate
glycerate3P	glycerate-3-phosphate

GMP	guanosine monophosphate
IMP	inosine monophosphate
NAcGlcnh2P	N-acetylglucosamine phosphate
pentoseP	pentose-phosphate
PEP	phosphoenolpyruvate
PPP	phosphoribosyl pyrophosphate
suc/2Mmal	succinate/2-methylmalonate
UDP	uridine diphosphate
UDP-AcGlcnh2	UDP-N-acetyl-D-glucosamine
UDPgal/UDPglc	UDP-galactose/UDP-glucose
UMP	uridine monophosphate
UTP	uridine triphosphate
